# Antagonistic evolution of an antibiotic and its molecular chaperone: how to maintain a vital ectosymbiosis in a highly fluctuating habitat

**DOI:** 10.1038/s41598-017-01626-2

**Published:** 2017-05-03

**Authors:** Claire Papot, François Massol, Didier Jollivet, Aurélie Tasiemski

**Affiliations:** 1 University Lille, CNRS, UMR 8198 - Evo-Eco-Paleo, SPICI group, F-59000 Lille, France; 2 AD2M, ABICE team, Université Pierre et Marie Curie-CNRS, UMR7144, Station Biologique de Roscoff, 29682 Roscoff, France

## Abstract

Evolution of antimicrobial peptides (AMPs) has been shown to be driven by recurrent duplications and balancing/positive selection in response to new or altered bacterial pathogens. We use *Alvinella pompejana*, the most eurythermal animal known on Earth, to decipher the selection patterns acting on AMP in an ecological rather than controlled infection approach. The preproalvinellacin multigenic family presents the uniqueness to encode a molecular chaperone (BRICHOS) together with an AMP (alvinellacin) that controls the vital ectosymbiosis of *Alvinella*. In stark contrast to what is observed in the context of the Red queen paradigm, we demonstrate that exhibiting a vital and highly conserved ecto-symbiosis in the face of thermal fluctuations has led to a peculiar selective trend promoting the adaptive diversification of the molecular chaperone of the AMP, but not of the AMP itself. Because BRICHOS stabilizes beta-stranded peptides, this polymorphism likely represents an eurythermal adaptation to stabilize the structure of alvinellacin, thus hinting at its efficiency to select and control the epibiosis across the range of temperatures experienced by the worm; Our results fill some knowledge gaps concerning the function of BRICHOS in invertebrates and offer perspectives for studying immune genes in an evolutionary ecological framework.

## Introduction

Antimicrobial peptides (AMPs) constitute key components of the innate immune system that rapidly eradicate or incapacitate pathogenic agents such as viruses, bacteria or fungi attempting to invade and proliferate multicellular eukaryotes^1–3^. In the last decade, they have also been shown to control and confine the symbiotic microflora in specific anatomical compartments (e.g. gut, bacteriomes, skin), thus contributing to the symbiostasis of both invertebrates and vertebrates^4–8^. In metazoans, the evolution of AMPs has been shown to be driven by recurrent duplications (i.e. creation of paralogs) and balancing/positive selection in response to new and/or altered bacterial pathogens that can be encountered in a novel habitat and/or that have rapidly evolved to escape the immune response^9–12^. In terms of co-evolutionary dynamics, patterns of evolution of AMPs thus seem to generally follow a hybrid route between the matching-allele (balancing selection at a given locus) and the gene-for-gene (arms race with pathogens through gene duplications with positive diversifying selection between paralogs) paradigms of Red Queen dynamics^13^. Most empirical evidence behind this assertion comes from experimentally challenged model organisms subjected to specific controlled conditions in the laboratory and/or from data focused on the well-protected inner part of the multicellular host *i.e*. the internal immunity (inside the body *sensu lato*). Because the body acts as a wall buffering external abiotic and biotic variations, selection processes driven by environmental constraints on innate immunity can be considered to fluctuate more outside the organism than inside.

Multiple data demonstrate that AMPs not only act internally but can also be secreted into the environment surrounding an organism where they participate in external immunity, referred to as “any heritable trait acting outside of an organism improving protection from pathogens or manipulating the composition of the microbial community in favor of an organism”^14^. In the case of extreme, frequently disturbed and stressful environments, the external immunity of an organism will depend on its ability to control the functioning of its externally secreted immune products under very variable conditions. In a sense, the coevolution of both the host immune system and the microbial communities in extreme environments adds another constraint to the usual Red Queen model, namely coevolution of two partners submitted to harsh selection for local adaptation to fluctuating environmental conditions, and this scenario has yet to be fully understood. Annelids are particularly suited to study the adaptation of external immunity to changing and harsh environmental conditions because (i) they are amongst the rare metazoans able to thrive in extreme and highly fluctuating habitats (e.g. hydrothermal vents, highly polluted anoxic sediments, polar environments), and (ii) they do not have barriers (i.e. exoskeleton or shell) to physically protect their skin from direct biotic/abiotic interactions. Rather than physical protection, they have developed a strong external immunity based on production of mucus and AMPs by the epidermic cells that respectively trap and kill/select pathogenic/symbiotic bacteria. In a sense, annelid defense is more comparable to that observed in amphibians or in mammals than that observed in cuticulates (i.e. arthropods and nematodes), the two most studied invertebrate phyla^8,15–17^. Polychaeta (marine worms considered as the primitive annelids) produce original AMPs, some of which are restricted to just one worm family (e.g. preproalvinellacin) or even a single species (e.g. preprohedistin). This suggests that a high AMP selection at the interspecific level has probably occurred in relation to the ecology of these organisms^18,19^.

In this study, we took advantage of the peculiar microbial and physico-chemical ecology of the extremophile annelid *Alvinella pompejana*, the most eurythermal and amongst the most thermo-tolerant animals known on Earth, to decipher the selection patterns acting on an AMP, namely alvinellacin, in an evolutionary ecological framework. By being part of the external immunity of *A. pompejana*, alvinellacin is at the direct interface with abiotic and biotic constraints imposed by life in the hottest part of the deep-sea hydrothermal ecosystem. Once secreted by the epidermal cells, alvinellacin accumulates on the surface of the worm and inside its tube, thus contributing to the external immunity of the worm^19^. Through its specific bactericidal activities, it participates in the control and selection of the environmental bacteria forming the typical complex symbiotic microflora that covers the dorsal tegument of this thermophilic annelid endemic of hydrothermal chimneys along the East Pacific Rise^19^. Epibionts have been shown to supply *A. pompejana* with nutrients and to detoxify heavy metals from their habitat^20^. The combination of this epibiosis with the particular microbial environment created inside the tube allows the worm to thrive under ‘hot’ conditions^21,22^. In its tube, *A. pompejana* actively pumps the surrounding cold seawater to bathe in a diluted mixed fluid, which is slightly less acidic and less concentrated in free hydrogen sulfides^23–26^. This behaviour exposes the consortium of Epsilon-proteobacteria making up the epibiota of *A. pompejana* to less extreme, but still fluctuating (ranging from 7° to 84 °C), environmental conditions^27^. According to an environmental genomic study, this peculiar habitat has led to the selection of a limited number of specialized bacterial strains with greater eurythermal adaptation and metabolic flexibility^28^. One intriguing point is how natural selection has operated on the worm’s external immunity to maintain this intimate and highly specific partnership present in all worms collected throughout its known geographic range (6,000 km)^28^.

In this context, the main goal of this study was to determine how external immune effectors, such as the *preproalvinellacin* gene, have been selected to maintain their efficiency at selecting and controlling the eurythermal epibiotic community in an extreme and fluctuating habitat. In order to understand the functioning of this immune gene as a controller of the worm’s epibiotic community, we examined (i) the levels of non-synonymous and synonymous genetic diversities over the different domains of the preproalvinellacin gene using two well-separated geographic populations of *A. pompejana*, and (ii) the divergence from its syntopic and phylogenetically close sister species *A. caudata* bearing the same epibiota. The originality of the present study lies in the search for the signature of adaptive evolution in an AMP (here alvinellacin) not in the context of pathogenicity, but rather in the context of the evolutionary constraints imposed by the obligatory maintenance of a specific, complex and vital ectosymbiosis in the face of eurythermality.

## Methods

### Specimen sampling

*Alvinella* spp. specimens were collected during the oceanographic cruises BIOSPEEDO (2004) and MESCAL (2012) at two geographically well separated sites (18°25.93S, 113°23.32W, 2640 m; 9°50.32N, 104°17.52W, 2550 m). Animals were collected with the manned submersible Nautile and directly flash-frozen on board (see Extended Experimental Procedures). DNA extractions were then performed using a CTAB/PVP protocol modified from^29^ and previously described in ref.^30^.

### PCR amplification, cloning and sequencing

The whole gene encoding the preproalvinellacin (1949 bp) was previously amplified by PCR using primers specifically designed from the 5′ and 3′ UTR regions^19^. Because of its length, the gene was then sub-divided into two regions for further amplification at the population level. A detailed description of the gene and the primer design is given in the Extended Experimental Procedures (Table S1). Allelic sequences were obtained from a series of individuals of the two *Alvinella* species using the mark-recapture cloning method^31^. A detailed description of the procedures together with the primer sequences are given in the extended methods (Table S1). Sequencing was run on an ABI 3100 using BigDye© v3.0 terminator chemistry and the retrieved sequences were proofread using the Geneious De Novo Assemble module. Alignments were then performed and adjusted using the PairWise/Multiple Alignment module of the Geneious software. *In vitro* recombinants due to cloning were manually checked by searching for any abnormal combination of tag ends and removed from the dataset. Sequence datasets were then cleaned for PCR-induced allelic chimeras and artifactual singletons following a complex procedure of recombinant removal using RDP4.0 (Recombination Detecting Program) software^32^. This procedure is described in detail in the extended experimental procedures.

### Paralog identification and individual genotyping

Because of duplications, a series of paralog-specific primers were designed from the cleaned sequence dataset without ‘natural’ recombinants (see Table S1). Allele genotyping within each locus was then performed on a subset of 16 individuals by direct sequencing of the 5′ region of the gene, in which there was enough diagnostic mutations to discriminate between the suspected paralogs (see extended experimental procedures).

### Genetic diversity and neutral tests

Standard molecular diversity indices (*S*, *θπ*, *θ*_*w*_, and *H*_*d*_) were estimated in *A. pompejana* for the 5′ region of the preproalvinellacin gene using the DnaSP v5.0 software^33^ globally and for each putative paralog, respectively. The estimators θ_w_ and θπ (both estimating the population parameter 4N_e_.µ under neutral assumptions) were compared to each other using Tajima’s D test and other neutrality tests such as Fu & Li’s D and Fu & Li’s F, which are more sensitive for detecting past demographic changes than Tajima’s D test^34^. The average number of nucleotide substitutions per site (D_xy_) was also computed between pairs of putative paralogs. Evolution of the multigenic and the within-duplicate genetic diversity (θπ) were also estimated along the gene for the two *Alvinella* species for both the exonic and intronic regions using a sliding window (size = 50 bp; step = 10 bp) with the software DNAsp 5.0^33^.

### Networks and coalescence trees

A phylogenetic reconstruction of duplications was performed on sequences obtained for the 3′ exonic region of the gene for two individuals of each species and for the 5′ region of the most-recaptured individuals of *A. pompejana*. The latter tree topology was then used to map amino-acid polymorphisms on the BRICHOS domain and to reconstruct the emergence of ‘natural’ recombinants. In both cases, the software jModelTest 2.1.7^35^ was used to select the best model of substitutions, and tree reconstructions were performed using the Maximum Likelihood method implemented in the software MEGA6^36^ and PhyML 3.0^37^ for the sake of comparison. The generalized time-reversible GTR+I+G model of substitutions^38–40^ was then tested against the selected best models, according to Akaike (AIC) and Bayesian (BIC) information criteria, using backward hierarchical likelihood ratio tests (hLRT), and subsequently used for the tree reconstruction (see extended experimental procedures). Allelic relationships (natural recombinants included) were examined in *A. pompejana* using the neighborNet method implemented in the program SplitsTree4 software package^41^ for the overall most divergent 5′ region only. This method was used as it uses reticulation to account for intergenic recombination.

### Search for positive selection along the preproalvinellacin gene

To detect selection imprints on each specific domain, a majority rule consensus sequence was built from each paralog domain to perform pairwise d_N_/d_S_ comparisons using the yn00 package of the PaML software^42^. Evolution of d_N_/d_S_ along the gene was also assessed by calculating both the average ratio of the fixed non-synonymous to synonymous substitutions, K_a_/K_s_, between paralogs and the average ratio of the polymorphic non-synonymous to synonymous substitutions, π_a_/π_s_, within each paralog using a sliding window (size = 50 bp, step = 10 bp) with the software DNAsp 5.0^33^.

### Mutation mapping on phylogenetic tree

Amino-acid replacements associated with the ‘hot spot’ of diversity in the BRICHOS domain were mapped onto the paralog ML tree topology obtained with the software MEGA 6.0. The ML tree was subsequently used as a reference to perform a likelihood (Empirical Bayes) reconstruction of ancestral amino-acid sequences using the aaML package of the PAML software with an empirical Dayhoff matrix of amino-acid replacements.

### Search for positive selection in the Propiece and BRICHOS regions

Alignments of the consensus coding sequences of paralogs of both the propiece region and its BRICHOS domain were used together with the outlier sequences of *A. caudata* to detect putative codons under positive selection using the Maximum Likelihood method implemented in the CodeML package of PaML, with the Goldman & Yang’s model of codon substitutions^43^. For both alignments, the software jModelTest 2.1.7 was used to select the best model of substitutions according to the AIC and BIC. This model was then used to reconstruct the reference tree topology using PhyML 3.0. The site models M1a, M2a, M7 and M8 were subsequently compared under the assumption of no variation in the mutation rate between duplicates over time (see extended experimental procedures)^43^. In addition, Bayesian methods (NEB and BEB) of codon classification into different classes of omega were also used with a p-value threshold of 0.95 to identify positively-selected sites. Only the BEB method is robust enough to separate positively-selected from selectively-relaxed sites without uncertainty.

### MacDonald-Kreitman test across paralogous genes

The MacDonald-Kreitman (MK) test was also used to detect signs of positive selection between pairs of paralogs for both the Propiece and BRICHOS domains taking advantage of their sequencing in several *A. pompejana* individuals using the module implemented in DNAsp vs 5.0. This test usually compares the ratios of non-synonymous and synonymous substitutions in the divergence (d_N_/d_S_) and in the polymorphism (p_N_/p_S_) of two closely related species but was also used to detect positive selection between pairs of paralogs^44–46^. The MK test was performed on a series of no recombining alleles to avoid excesses of non-synonymous polymorphic changes by recombination and loss of power in detecting positive selection in the paralog divergence. We also checked whether one of the assumptions of the MK test may be violated by a possible selective relaxation prior to the duplication event (see ref.^47^) by performing a branch-model comparative analysis of duplicates (i.e. the “one ω ratio” M_0_ vs. the “free ω ratio” M_1_) using the package CodeML of the software PaML^42^.

### Preproalvinellacin gene induction in animals exposed to different thermal and pressure regimes

Experiments were performed onboard during the MESCAL cruise. For the pressure experiments, *Alvinella* individuals (n = 10) were transferred immediately after raising from 2500 m into the DESEARES aquarium and maintained at 250 bars for 12 h to recover from depressurization, at constant temperature. A thermal shock experiment was also performed with a new set of isobaric BALIST equipment to retrieve and conduct experiments on worms at a constant *in situ* pressure^48^. Briefly, after recovery from the PERISCOP sampling device, *A. pompejana* specimens (n = 9) were subjected in the BALIST aquarium to three distinct thermal shocks (20, 42 and 54 °C) for 2 hours. Expression of the inducible hsp 70 and preproalvinellacin genes was quantified by RTqPCR from total RNA extracted from the experimental specimens according to the procedures detailed in ref.^48^. Gene expression was normalized to expression of RPS26.

## Results

### Gene diversification of preproalvinellacin in the genus *Alvinella*

**Figure 1:**
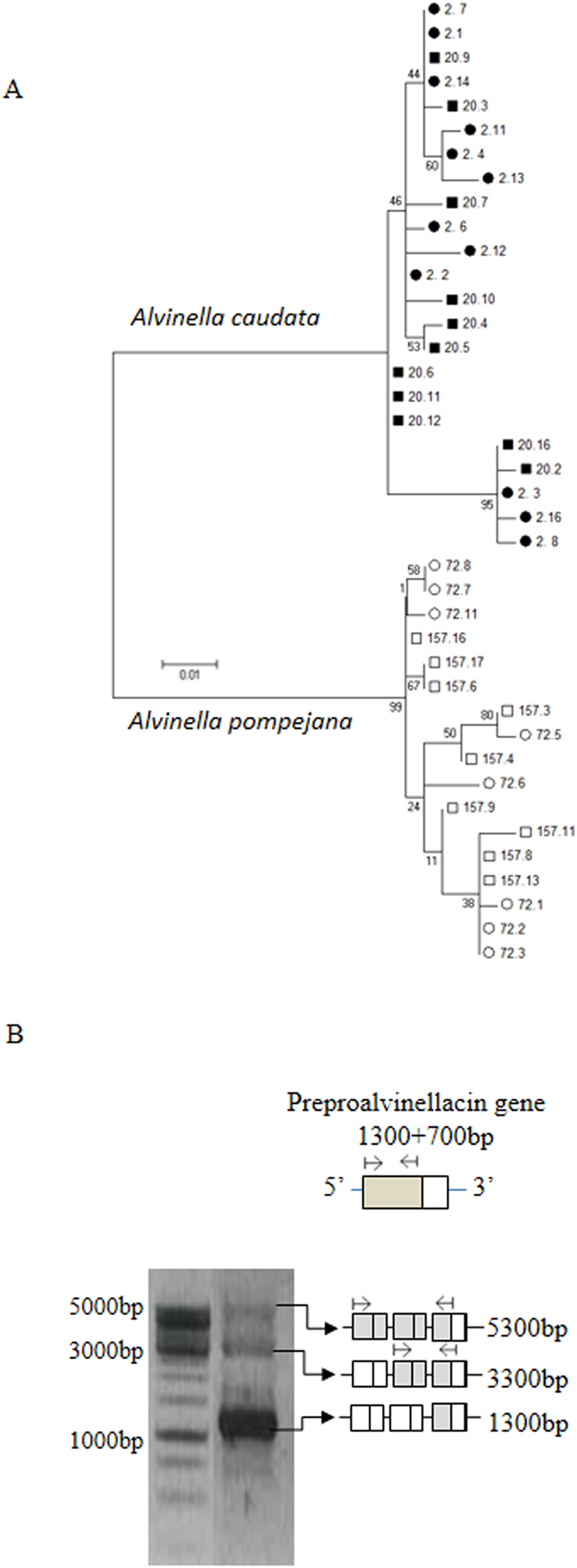
Gene diversification of preproalvinellacin. (**A**) Coalescence tree of alleles found in two well-recaptured individuals of *Alvinella pompejan*a (white) and its sister species *Alvinella caudata* (black), and (**B**) molecular evidence that it comes from tandemly repeated gene duplications in *A. caudata*. (**A**) Reconstruction of lineages without recombination was performed on the 3′ region on all nucleotide sites by Maximum Likelihood using the K2P model in MEGA 5.0. Allele coverage: A. *caudata*: two individuals with 15 clone recaptures each, *A. pompejana*: two individuals with 50 clone recaptures each. (**B**) Agarose gel electrophoresis with a 1 kb DNA ladder showing the amplification of the 1300 bp 5′ region of the *A. caudata* preproalvinellacin (complete gene: 2000 bp) using the 5′ primers (arrows). Longer extra bands indicate the co-amplification of two and three linked genes as summarized by the boxes representing the tandemly-duplicated gene and the position of the PCR products.

A first allelic screening revealed that the number of alleles per individual greatly exceeded two for both *Alvinella* species and ranged from 4 to 12 alleles according to the effort of recapture, even after correcting for singleton excesses. After removing PCR-artifactual recombinants between heterozygous gene copies, a phylogenetic reconstruction of alleles was performed using the four most recaptured individuals of the two *Alvinella* species (Fig. 1A and Fig. S1). The resulting tree displayed a reciprocal monophyly between the two species with a rather flattened shape of the coalescent, suggesting a recent and independent diversification of the gene after speciation (Fig. 1A). In both species, alleles were grouped in more than two clades for each of the four individuals tested. Tandem duplication was then supported by the size of the PCR products obtained on genomic DNA amplified with the forward and reverse preproalvinellacin primers (Fig. 1B).

**Figure 2:**
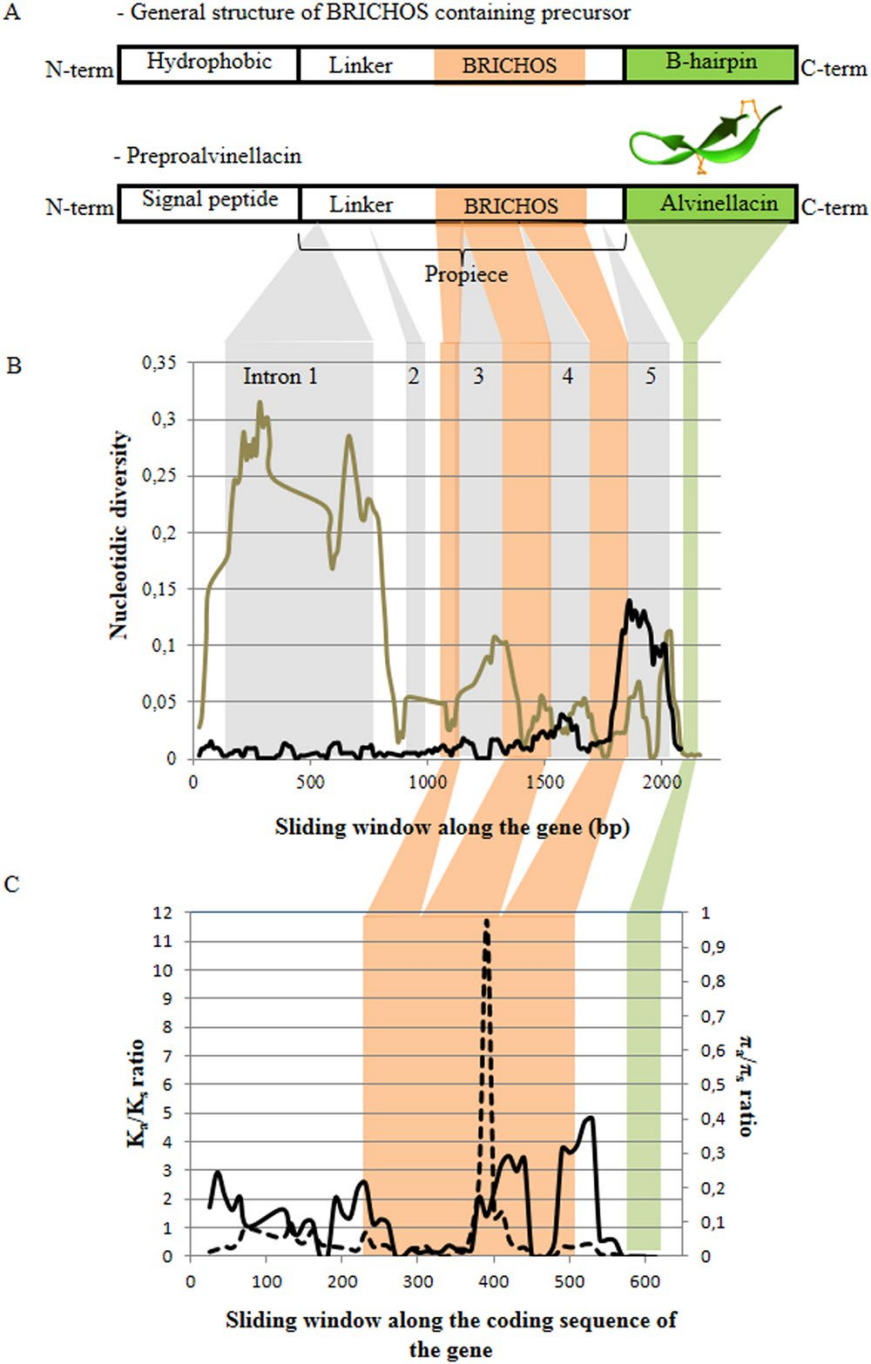
Evolution of genetic diversity and K_a_/K_s_ along the preproalvinellacin gene and its corresponding coding sequence: (**A**) active alvinellacin is cleaved from a larger proteic precursor (*i.e*. preproalvinellacin). In contrast to all described AMPs, the preproalvinellacin family harbors the pattern of a BRICHOS containing protein: a hydrophobic domain (the signal peptide), a propiece with a linker and a BRICHOS domain and a C-terminal region with β-sheet propensities (alvinellacin). (**B**) Sliding window of the overall nucleotidic diversity (θπ) along the intronic and exonic regions of the gene in *A. pompejana* (black) and *A. caudata* (grey), (**C**) between-paralog K_a_/K_s_ mean (dashed line/left) and the average within-paralog π_a_/π_s_ (solid line/right) along the coding sequence of the gene. Sliding window length = 50 bp, step size = 10 bp. Introns are colored in grey (5 introns); exons are colored as follows: orange: BRICHOS domain, green: AMP domain.

Global genetic diversities were similar for the two species, but strikingly differed in intensity along the precursor gene (Fig. 2A,B). In *A. pompejana*, the gene displayed an astonishingly high nucleotidic diversity (θπ = 0.25) in the first intron, whereas it only reached a maximum (around 0.15) in the last intron preceding the AMP coding region in *A. caudata*. Both species displayed lower genetic diversity in exons than in introns with similar levels of variation (θπ = 0.025). The last exon, containing the AMP, was unexpectedly monomorphic in *A. pompejana* and weakly polymorphic (with three distinct variants) in *A. caudata* (Fig. S1). Finally, the two *Alvinella* AMPs differed by only one fixed replacement (Ser - > Asp) at position 198 (Fig. S1).

### Identification and characterization of paralogous genes in *A. pompejana*

**Figure 3:**
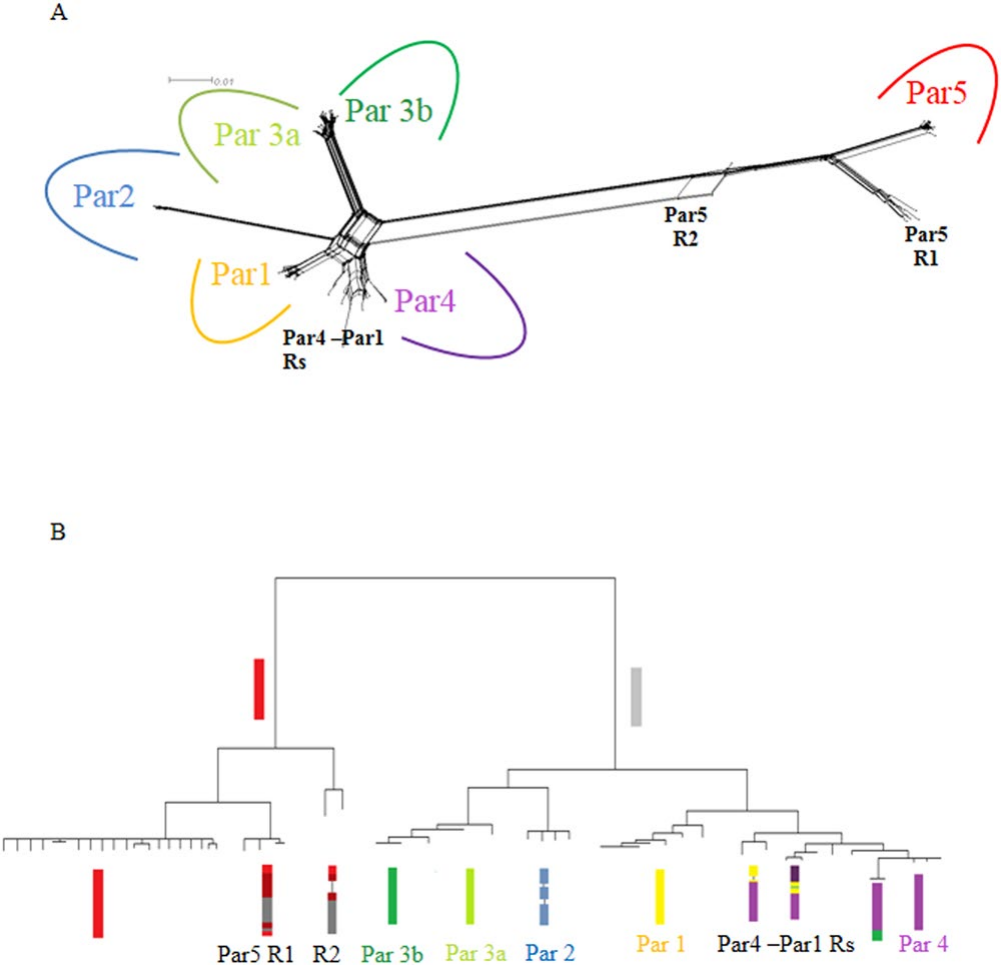
Splits Tree reticulated network of all alleles encoded by the preproalvinellacin multigenic family in *A. pompejana:* (**A**) the NeigborNet network together with (**B**) the schematic reconstruction of natural intergenic recombinants using the NJ tree topology obtained with the MEGA 5 software and the HKY model of substitutions. Sequences from the 5′ region were used in the two reconstruction methods and the six paralogs (Parx) as well as their intergenic recombinants (Rx) are represented by boxes in which the red, dark green, light green, blue, yellow and purple colors represent the genetic paralogous background and their proportions in recombinants, darker colors, the portions of the recombining alleles which accumulated their own set of mutations, and bars correspond to indel polymorphisms (including alleles with the 34 codons deletion in Par5).

The genotyping of individuals with paralog-specific primers allowed us to distinguish four paralogs (1, 2, 4 and 5) with either a homozygous or heterozygous state for each individual. Paralog 3 displayed more than 3 alleles per individual and was subsequently sub-divided into par3a and par3b. Once this genotyping procedure had been performed, the sequence dataset was assembled as a reticulated network of alleles (Fig. 3A) and led to the same exact ML tree topologies (Fig. 3B) using either the GTR+I+G or the selected best models of substitutions obtained from the Bayesian informative criterion (BIC) of jModelTest (Tables S2 and S3, Fig. S2). This network, together with the distribution of alleles within each individual, indicated that preproalvinellacin is encoded by a multigenic family of at least six genes. Five distinct recombination events were robustly characterized (Fig. 3B), each of these displaying a different series of linked sites (mainly in the intronic parts of the gene) in several individuals. Three of these recombinants displayed their own set of specific mutations and represent ‘old’ events in the history of diversification of the gene. The number of nucleotide substitutions per site (D_xy_) was computed between pairs of paralogs, leading to an average divergence of 0.158 (Table S4). Without the most divergent paralog par5 (D_xy_ = 0.314), the remaining divergence for the other pairwise comparisons was four times smaller (D_xy_ = 0.079). Both nucleotide (θπ) and haplotype (H_d_) diversities were high and quite variable between paralogs but did not significantly differ from neutral expectations (Table 1).

**Table 1:** Genetic diversities and associated statistical tests for the six preproalvinellacin paralogs of *Alvinella pompejana*.

	N	L	S(ex)	Nsites (ex)	Pi exon	Nsites (ex+int)	S (ex+int)	pi (ex+int)	ThetaW	Hd	Tajima	Fu&Li F	Fu & Li D
par1	12	1293	10	315	0.0078	978	25	0.0048	0,0064	0.985	−1.145	−1.218	−1.365
par2	6	1124	2	214	0.0029	910	8	0.0026	0,0031	1	−1.072	−1.063	−1.145
par3a	13	1242	8	315	0.0058	927	30	0.0059	0,0078	1	−1.047	−0.998	−1.157
par3b	9	1239	8	316	0.0055	923	22	0.0055	0,0065	1	−0.804	−0.674	−0.791
par4	14	1114	12	316	0.0112	798	54	0.0121	0,0158	0.978	−1.039	−0.617	−0.842
par5	25	1270	8	349	0.0024	921	37	0.0032	0,0077	0.983	−2.207**	−3.360**	−3.518**

N: number of sequences; L: length of the paralog. S: number of polymorphic sites in both exonic (ex) and entire region (ex+int), Nsites: effective length of each domain, Pi: nucleotide diversity, ThetaW: Theta Watterson, Hd: Haplotypic diversity, Tajima: Tajima’s D test, Fu&Li F: Fu and Li’s F test, Fu&Li D: Fu and Li’s D test. Statistical significance: ** for *P* < 0.05.

### Strength of selection between domains and along the gene

Domains exhibited striking differences in terms of K_a_/K_s_ ratios between paralogs (Fig. 2, Table S5). In the signal peptide, nearly all paralogs exhibited the same amino-acid signature with the exception of par1. Values of K_a_/K_s_ were more heterogeneous in the propiece region and the BRICHOS domain with ratios close to or exceeding one between many pairs of paralogs, including the most divergent paralog 5/E in the BRICHOS domain. By contrast, the AMP itself displayed no K_a_/K_s_ signal because of its lack of genetic variation.

A sharp peak of K_a_/K_s_ between positions 360 and 410 was observed in the BRICHOS domain with a maximal value of up to 12 when looking at the polymorphism-to-divergence variation along the gene using a sliding window (Fig. 2C). In contrast, the averaged π_a_/π_s_ across paralogs exhibited several peaks in both the peptide signal and the BRICHOS domain, but with maxima well below one, suggesting diversifying selection between the duplicated genes (Fig. 2C). McDonald-Kreitman tests failed to detect significant positive selection between pairs of paralogs either in the 5′ or the 3′ regions of the gene. Such a failure was possibly due to the high recombining rate between paralogs even if the alignments tested were devoid of recombining alleles.

### Ancestral reconstruction of the BRICHOS domain and mutation mapping

**Figure 4:**
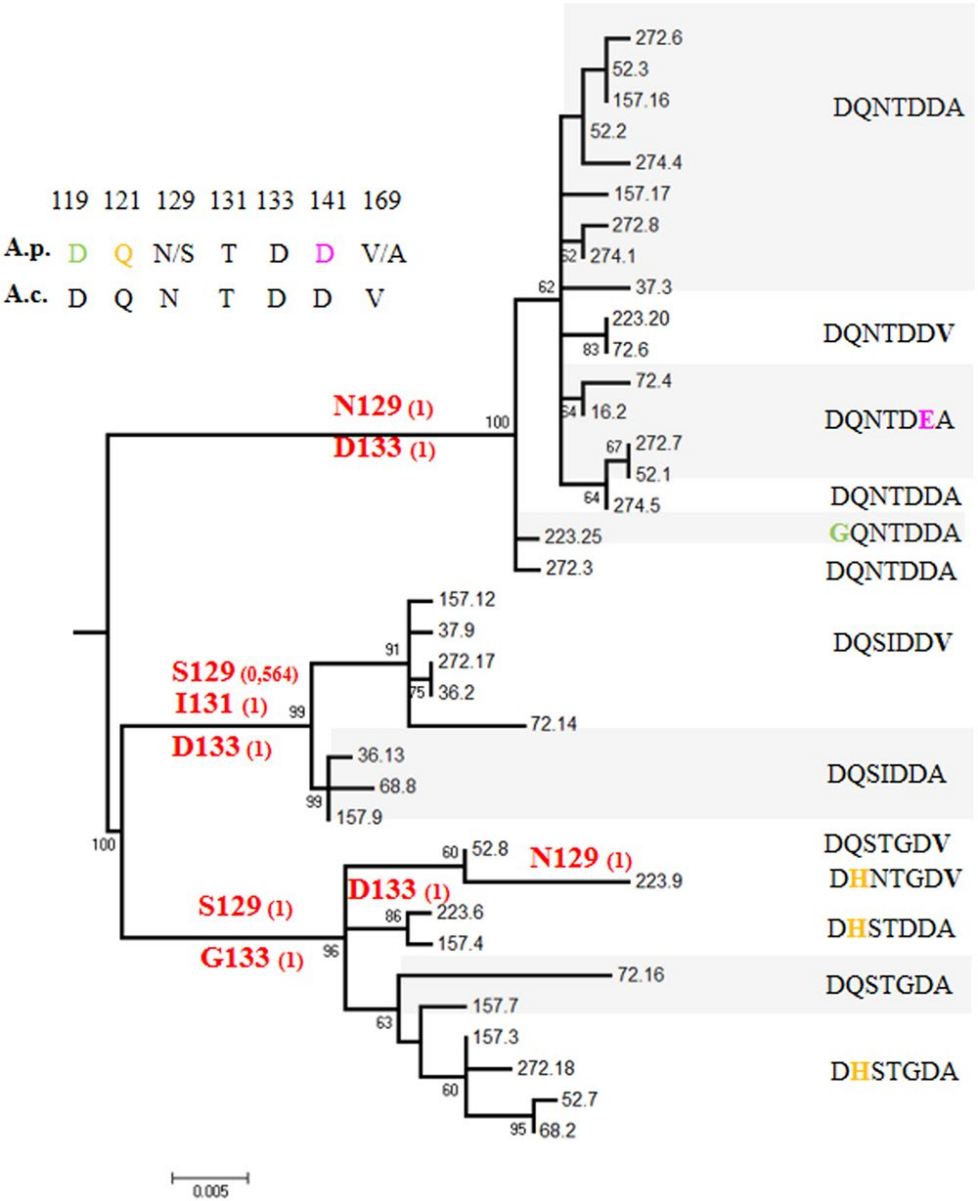
Ancestral reconstruction of polymorphic amino-acid replacements in the BRICHOS domain and the mature AMP in *Alvinella pompejana* (Ap) using the aaML package of PaML4.0 and *Alvinella caudata* (Ac) as an outgroup to orientate mutations. Positions of amino-acid replacements are labeled according to the start (methionine) codon. The Bayesian probability of occurrence of an amino-acid in the ancestral sequence is given in brackets. The tree topology and associated bootstrap values (obtained from 1000 replicates) was obtained by the ML method in Mega 5.0 using the full sequences of the 3′ region of the gene. Sequence labels represent the individual number and the clone number and are representative of the 6 paralogous clades (excluding natural recombinants) and subsequently used in the mapping of the BRICHOS mutations (see BRICHOS amino-acid alignment in Fig. S1).

Both *Alvinella* species exhibit a high number of amino-acid replacements over a small portion of the BRICHOS domain, but on distinct sites. The ancestral reconstruction of 36 BRICHOS sequences allowed us to infer whether the ‘hot spot’ of non-synonymous diversity was likely due to the retention of an ancestral polymorphism or to the positive fixation of some specific mutations between duplicates. At least seven polymorphic amino-acid replacements were found in the BRICHOS domain (Fig. 4). Reconstructions at ancestral nodes leading to duplicates were robust (p > 0.99) and indicated that three of them (N129S, T131I and D133G) were paralog-diagnostic. Reconstruction of ancestral states was similar when applying the same method on a smaller set of consensus coding sequences of duplicates using the selected best model in jModelTest (K80+I model, Fig. S3). Other replacements were randomly distributed between paralogs in terminal positions, suggesting either that these mutations reflect an ancestral polymorphism or have recently been exchanged by recombination. In the Propiece region, we evidenced at least 20 sites of amino-acid replacements among paralogs. Both the GTR+I+G model and the selected best substitution model led to the exact same tree topology among Propiece paralogs (Fig. S4).

### Search for positively-selected codon sites in the propiece including the BRICHOS domain

Search for positively-selected sites was performed on a restricted dataset of 8 consensus sequences (Table 2), but also over all the recovered non-recombining alleles (79 sequences for the propiece and 36 sequences for the BRICHOS domain, see extended experimental procedures, Tables S6 and S7). Comparing the free-ratio Branch model (M_1_) with the one-ratio Branch model (M_0_) did not produce significant differences in goodness-of-fit for either the BRICHOS (LRT = 9.44, df = 13, NS) or the propiece (LRT = 19.80, df = 13, NS) domains, suggesting that non-synonymous changes may be equally distributed among branches of the paralogous tree. The LRT was also not significant when comparing the no-clock model with the model assuming a global molecular clock for both domains, suggesting that the substitution rates may have been constant over time during duplications. Likelihood ratio tests performed between Site models using the consensus sequence dataset were significant for the propiece region (p-value < 0.01), not the BRICHOS. This finding was also supported by analyses in which polymorphic sequences were also included for the two genic regions under scrutiny (Tables S6 and S7). The comparison between models M_0_ and M_3_ indicated that ω is heterogeneously distributed among codon sites along the two domains (Table S6). Other comparisons (M_1a_ vs. M_2a_; M_7_ vs. M_8_) went a step further and gave credence to the hypothesis of positive selection with a non-negligible proportion of the codon sites under diversifying selection for the propiece region only (34%, ω = 4.84; Table 2). Eight positively selected sites were identified in the BRICHOS domain using the Naive Empirical Bayes (NEB) method, some of which were previously identified in Figs 4 and S1. None of these remained significant with the BEB method (Table 2). In the propiece domain, fifteen codon sites exhibited a significant NEB probability, with two still significant with the BEB method under the M8 model (sites P60Y* and Q68E*). Results were nearly similar with all polymorphic sequences (Tables S6 and S7).

**Table 2:** Log-likelihood values and parameter estimates for the BRICHOS and propiece domains of the preproalvinellacin gene using models implemented in the CodeML program of the PAML package with the alignments of consensus paralogous sequences (8) and the reference tree selected by jModelTest 2.1.7 (BRICHOS: K80+I, Propiece: Tim2ef I+G).

BRICHOS	Branch Models				Site Models			
	Model	M0	M0 (with clock)	M1 (free-ratio)	M1a	M2a	M7	M8
	**Log likelihood**	−375.37	−381.68	−370.65	−374.63	−374.33	−374.63	−374.33
	**Parameters**	ω = 0,612, kappa = 14.4	ω = 0,625, kappa = 14.4	8 branches with ω > 1, kappa = 14.3	(ω_0_ = 0) p_0_ = 0.49, (ω_1_ = 1) p_1_ = 0.51, kappa = 13.9	(ω_0_ = 0) p_0_ = 0.67, (ω_1_ = 1) p_1_ = 0, (ω_2_ = 1.92) p_2_ = 0.33	p = 0.0050, q = 0.0052, kappa = 13.8	p_0_ = 0.67 (ω_0_ = 0), p = 0.005, p_1_ = 0.33 (ω = 1.92) q = 1.75
	**Sites with dN/dS > 1 (NEB analysis)**	n.a.	n.a.	n.a	n.a.	(8 sites**) D119G; Q121H; R124R; N129S; T131I; D133G; D141E; V169A	n.a.	(8 sites**) D119G; Q121H; R124R; N129S; T131I; D133G; D141E; V169A
	**Sites with dN/dS > 1 (BEB analysis)**	n.a.	n.a.	n.a.	n.a.	V169A (ns)	n.a.	N129S; V169A (ns)
	**LRT**		12.62^NS^ (df = 7)	9.44^NS^ (df = 13)		0.6^NS^ (df = 2)		0.6^NS^ (df = 2)
**PROPIECE**	**Branch Model**				**Site Model**			
	**Model**	**M0**	**M0 (with clock)**	**M1 (free-ratio)**	**M1a**	**M2a**	**M7**	**M8**
	**Log likelihood**	−406.49	−412.34	−396.57	−405.02	−400.41	−405.02	−400.41
	**Parameters**	ω = 1.179, kappa = 9.2	ω = 1.189, kappa = 9.3	8 branches with ω > 1, kappa = 9.3	p_0_ = 0.39 (ω_0_ = 0), p_1_ = 0.61 (ω_1_ = 1)	p_0_ = 0.66 (ω_0_ = 0), p_1_ = 0 (ω_1_ = 1), p_2_ = 0.34 (ω_2_ = 4.84)	p = 0.009, q = 0.005, kappa = 7.1	p_0_ = 0.66 (ω_0_ = 0) p = 0.005, p_1_ = 0.34 (ω = 4.84) q = 12.52
	**Sites with dN/dS > 1 (NEB analysis)**	n.a.	n.a.	n.a.	n.a.	(15 sites**) M22I: W24R; L26Q; N30S; A31V; H33D; I36T; E37K; P38Y; D57E; T60I; Q68E; D72N; H77R; L78S	n.a.	(15 sites**) M22I: W24R; L26Q; N30S; A31V; H33D; I36T; E37K; P38Y; D57E; T60I; Q68E; D72N; H77R; L78S
	**Sites with dN/dS > 1 (BEB analysis)**	n.a.	n.a.	n.a.	n.a.	(4 sites^$^ with p > 0.85) M22I: W24R; L26Q; N30S; A31V; H33D; I36T; P38Y^$^; D57E; T60I^$^; Q68E^$^; H77R^$^; L78S	n.a.	(4 sites^$^ with p > 0.85 and 2 sites*) M22I: W24R; L26Q; N30S^$^; A31V^$^; H33D; I36T; P38Y^*^; D57E; T60I^$^; Q68E^*^; H77R^$^; L78S
	**LRT**		11.70^NS^ (df = 7)	19.84^NS^ (df = 13)		9.22** (df = 2)		9.22** (df = 2)

M0 (one-ratio); M1 (free-ratio); M1a (nearly-neutral); M2a (selection); M7 (β); M8 (β+ω > 1) and the estimated log-likelihood values (l) by the CodeML program, ω = dN/dS nonsynonymous/synonymous rate ratio; p = proportion of sites for each site class. M0: one ω for the tree; M1: one ω per branch, M1a: p_0_ = proportion of sites with ω_0_ = 0, p_1_ = 1 − p_0_, proportion of sites with ω_1_ = 1; M2a: p_0_ (ω_0_ = 0), p_1_ (ω_0_ = 1), and ω_2_, p_2_ = 1 − p_0_ − p_1_. M7: p and q (parameters of β distribution of ω between 0 and 1). M8: same as M7 except the addition of one site class in which ω is greater than one. Positively Selected Sites: Codon positions predicted to be under positive selection with a posterior probability of acceptance of ^$^>85%, *>95% and **>0.99 (identification of sites exhibiting dN/dS ratio > 1) with either Naive Empirical Bayes (NEB) or Bayesian Empirical Bayes (BEB). Numbers refer to amino-acid positions from the initial methionine. LRT: likelihood ratio tests between substitution models (between nested branch models: M0 vs. M0 with clock, M0 with clock vs. M1; between nested site models: M1a vs. M2a, M7 vs. M8). NS: not significant, **p < 0.05.

### Preproalvinellacin gene induction upon abiotic stress

**Figure 5:**
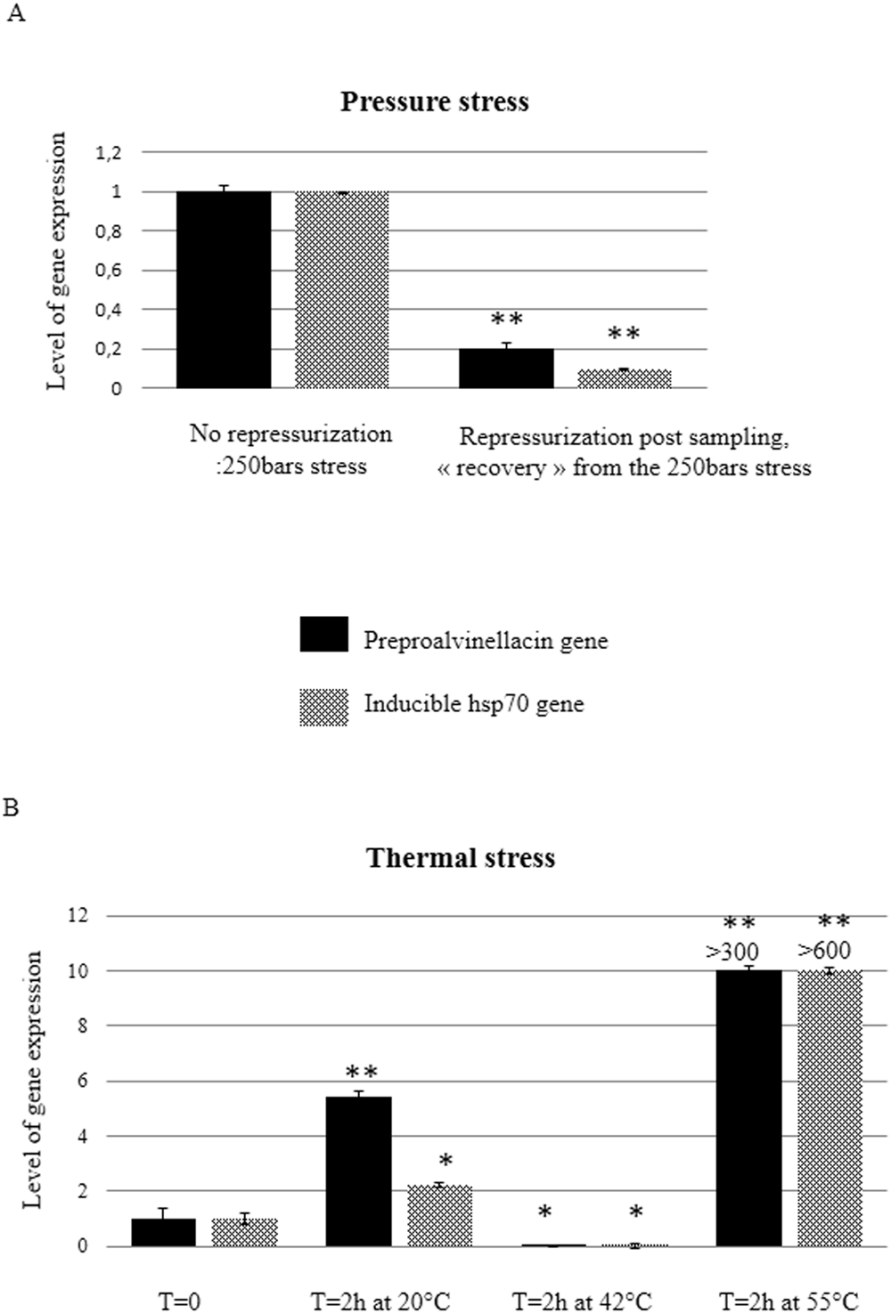
Upregulation of expression of the preproalvinellacin gene and the molecular chaperone hsp 70 in *Alvinella pompejana* submitted to thermal or pressure stress. (**A**) The level of transcription is significantly higher for both genes in “not re-pressurized animals” compared to those re-pressurized immediately after raising from −2500 m at the *in situ* pressure of 250 bars in the DESEARES vessel. (**B**) In animals kept under *in situ* pressure in the BALIST device, thermal stress led to an up-regulation of the two genes. P-values from Student’s tests were calculated *versus* the control treatment (normalized to 1), based on the experimental measures performed in triplicates (**p < 0.01, *p < 0.05).

The level of expression of the BRICHOS containing preproalvinellacin gene was evaluated under two stressful conditions previously shown to induce the synthesis of molecular chaperones (Heat shock proteins, Hsp) in *Alvinella pompejana* ^48^. Both the preproalvinellacin and the Hsp70, quantified by RT-qPCR, produced exactly the same pattern of gene expression in worms submitted to various temperatures and pressure stresses (Fig. 5). This data supports the conclusion that the *Alvinella* BRICHOS, in addition to its sequence similarities, behaves like a molecular chaperone.

## Discussion

The evolution of a newly described AMP gene involved in the external immunity of annelids was investigated in two sister alvinellid species. This AMP participates in maintaining a highly conserved ecto-symbiotic microflora vital to life in the highly fluctuating vent habitat. In order to explain the lack of genetic diversity of the AMP itself in spite of a very high level of gene diversification, signs of positive selection were sought within the different functional domains of the propiece. The roles of the BRICHOS chaperone and the alvinellacin AMP have been teased apart in the context of the abrupt thermal variations encountered by the worm.

### ‘Hot spots’ of high genetic diversity result from tandem duplications and intergenic recombination

The role of duplication in the diversification of AMP genes has been well documented, leading to complex multigenic families^49–52^. This process may itself be adaptive in the co-evolutionary arms race between the host and pathogens by generating new copies of AMPs able to evolve more rapidly, and thus displaying new antimicrobial properties against newly encountered microbes, without erasing old functions^9^. Our genetic dataset demonstrates that the preproalvinellacin peptide follows this rule and is encoded by a multigenic family of at least six genes, some of which are repeated in tandem. Reciprocal monophyly of the coalescence trees combined with the non-juxtaposition of the intronic ‘hot spots’ of diversity between the two *Alvinella* sister species indicates that the diversification of the preproalvinellacin gene occurred independently through recurrent and recent duplication events after speciation. The two *Alvinella* species separated a long time ago (several tens of millions of years) with about 23% divergence on the mtCOI^53^. Independent and recent duplications have already been reported, e.g. in murine beta-defensins for which gene duplications took place after mice and rats diverged ca. 40 million years ago^54^. Consequently, these *Alvinella* species can be considered as ecological replicates of the same symbiotic model in which independent duplications arose probably in response to the gradual appearance of epibiosis, suggesting that the ancestor was not fully equipped to sustain durable interactions with existing microbial communities. Tandem duplications seem to have been the most appropriate mechanism for the long-term acquisition of a strict complex multi-specific epibiosis.

Series of intra- and inter-genic ‘natural’ recombinants were also identified, mainly in the first part of the preproalvinellacin gene without exon shuffling or deleterious frame-shifts of the coding sequence. Natural intergenic recombinants are rarely present in tandemly duplicated genes because of concerted evolution^55^. In the immune system, however, recombination seems to play a positive role in producing allelic diversity as previously shown for MHC class I genes^56^. Successful recombination events have been frequent in the preproalvinellacin gene since the first duplication event, with some early duplications subsequently kept in populations and now displaying their own pattern of accumulated mutations (0.052 substitution per site). Mutation and recombination are two major evolutionary mechanisms driving genetic diversity and likely to promote an adaptive response to cope with biotic interactions, but their relative contribution varies greatly between genes and organisms^57^. In the specific case of preproalvinellacin, the main positive outcome of intergenic recombination would be the spread of positively selected mutations between duplicates leading to the observed patterns of “shared polymorphism” in the propiece and BRICHOS domains between paralogs. Although we cannot rule out the hypothesis of retention of an ancestral polymorphism due to balancing selection occurring within duplicated genes, a more likely explanation would be that the observed mutation reversals are the result of a transfer of newly gained positive mutations from one duplicated gene to another via gene conversion or unequal crossing-over as previously proposed in newly duplicated genes in tandem^58^. Balancing selection within duplicates would have led to high π_a_/π_s_ ratios around the non-synonymous shared polymorphisms, a situation not recorded here.

### An antimicrobial function under strong purifying selection

The alvinellacin AMP is strictly monomorphic and identical between paralogs. No variation has even been observed between individuals issued from geographically-disjoint populations of *A. pompejana* situated on either side of the equator and thus physically separated since at least two million years^59^. AMP sequences of the two *Alvinella* species diverge only by one amino-acid replacement (N- > S) despite speciation having occurred a long time ago^53,60^. Thus, a strong directional purifying selection must have occurred on the antimicrobial effector. The strong purifying selection acting on the antimicrobial function is probably a consequence of a convergent evolution of the two species to the same microbial environment (at least one dominant ε-proteo phylobiont shared between species^20^). The two *Alvinella* species live syntopically on the walls of high-temperature chimneys along the East-Pacific Rise. This strongly suggests that host-symbiont interactions have co-evolved very slowly, possibly due to the high level of specialization of the vent microflora since the beginning of cretaceous, despite the highly chaotic variations of vent fluid discharge^61^. By contrast, changing/fluctuating biotic conditions, such as those exerted by rapidly co-evolving parasites, often result in signs of balancing selection, thus contributing to the maintenance of AMP polymorphisms as observed in arthropods^11,62^. Such patterns of purifying selection have also been observed in defensin genes of primates, which are associated with function(s) that became fixed quite early in mammalian evolution, making them less variable than expected under neutral evolution^63^. More generally, the evolution of AMPs appears to be a slow process compared to the situation observed for other immune-related genes^62,64^. It is remarkable and puzzling that AMPs are so well conserved at the species level and maintain their activity against co-evolving microbes, especially against symbionts that could have developed cheater strategies after such a long co-evolutionary time. One hypothesis is that AMPs act in complex combinations that can be synergistic, making the establishment of microbial resistance against a particular AMP difficult^62,65–69^.

### The BRICHOS domain prevents the incorrect folding of alvinellacin as temperature fluctuates

It is of particular interest that *A. pompejana*, which is still considered as one of the most thermotolerant and eurythermal animals on Earth, synthesizes a precursor containing an AMP with β sheet structure (alvinellacin) together with a molecular chaperone (BRICHOS). The BRICHOS domain constitutes the first example of a chaperone-like domain with a high propensity and specificity for β-prone regions^70,71^. In mammals, this chaperone binds with β hairpin motifs in order to prevent β sheet aggregation and amyloid fibril formation, which is highly toxic for organisms as exemplified in various diseases such as Alzheimers syndrome^72^. As such, this domain ensures correct β sheet folding and the subsequent activity of the protein released from the same precursor. Mutations in this domain are causative agents of multiple diseases e.g. stomach cancer, dementia, and respiratory distress in humans^73^. Even though a BRICHOS domain of over 300 proteins from 12 different protein families was identified in both protostomian and deuterostomian models in 2009^74^, its association with an AMP constitutes the first observation of its direct implicit folding function in an invertebrate.

There is still no experimental method to simply assign a molecular chaperone function to a protein: the strongest presumption that a protein plays this ‘repairing’ role remains, even today, its induction upon stressful conditions. Our data show a 350-fold up-regulation of the gene encoding the preproalvinellacin precursor with increasing thermal shocks, thus supporting the hypothesis of its chaperone function in addition to its sequence homology with BRICHOS. Interestingly, BRICHOS and alvinellacin are both released from the epidermis cells into the acidic and thermally highly fluctuating environment of the Pompeii worm. These conditions typically favor the auto-aggregation and accumulation of molecules having a β sheet tertiary structure (like alvinellacin), generating (i) toxic amyloid fibrils, and (ii) loss of the antimicrobial properties of the molecule. Consequently, BRICHOS presumably ensures the correct folding of the secreted AMP over a wide range of thermal conditions and thus the optimal functioning of the AMP to shape and control the vital ectosymbiosis. As such, it could be a likely target for positive diversifying selection to cope with this extremely variable thermal habitat.

### Evolutionary dynamics of the preproalvinellacin gene

Maximum likelihood ratio tests (CodeML) and both neutral (Tajima’s D) and ‘selection’ (McDonald-Kreitman) tests were performed to determine whether evolution of the preproalvinellacin multigenic family is driven by positive selection and whether selection acts differentially on the subsequent functional domains of the propiece region. Values of both θπ and d_N_/d_S_ along the gene indicate the occurrence of an unexpected very sharp ‘hot spot’ of non-synonymous mutations on the BRICHOS domain. Mapping these variants on the tree of paralogs suggests that this domain duplication was followed by a positive diversifying selection. Unlike the propiece region, the MK tests and our likelihood/Bayesian tests fail to discriminate positive selection from selective relaxation in the chaperone domain of the gene, probably as a consequence of the high rate of recombination between paralogs in this specific region. Overall, our results highlight a complex selective situation in which duplicates have first been subjected to diversifying selection (*i.e*. positive diversification by duplication: see results on the propiece domain), then partially compensated by sporadic genetic exchanges due to gene conversion/recombination, which in turn have acted in order to maximize genetic diversity over the whole set of these tandemly repeated genes (*i.e*. maintenance of the whole system by balancing selection). In contrast to the alvinellacin peptide, the high number of non-synonymous variants of BRICHOS may reflect a response to selective environmental constraints that act specifically on this region. Adaptation to high temperatures is a complex evolutionary process that can involve modifications of the intrinsic stability of proteins^75^ and/or interactions with molecular chaperones that help stabilize or re-fold the focal protein. Here, it appears that adaptation to highly fluctuating temperatures has acted more specifically on the propiece region and its molecular chaperone. As the conformation of a molecule is key to its biological activity, we hypothesize that the observed allelic variants of BRICHOS contribute to the stabilization of the alvinellacin hairpin in the context of the variable abiotic (thermal) conditions of the tube habitat, thus maintaining an efficient external immunity against pathogenic bacteria and an efficient control of vital epibiota.

## Conclusion

Highly fluctuating physico-chemical conditions have not promoted diversifying selection on alvinellacin *per se* in contrast to the situation generally observed in other metazoan AMPs. On the contrary, a strong purifying selection is evident, despite the duplication-driven diversification of its chaperone containing precursor. Duplication of genes has often been viewed as a molecular mechanism by which animals or plants adapt to changing environmental conditions with little cost^76^. Here, we demonstrate that exhibiting a vital and highly conserved ecto-symbiosis in the face of thermal fluctuations has led to a peculiar selective trend promoting the adaptive diversification of the molecular chaperone of the AMP, but not of the AMP itself. This finding significantly differs from previous results, as no polymorphism (following the “matching allele” model of Red Queen theory), nor duplication and ensuing divergence (following the “gene-for-gene” model) was observed for alvinellacin. As a consequence, because of the uniqueness of its chaperone, the preproalvinellacin gene family represents an interesting model to better understand the evolution of external immunity *in natura*. Our results fill some knowledge gaps concerning the function of BRICHOS and revive innovative topics that question the evolutionary success of the BRICHOS domain in a large variety of animal proteins, notably its anti-amyloid function which may have appeared early in the history of life.

## Electronic supplementary material

SUPPLEMENTARY INFORMATION

## References

[1] Zasloff M (2002). Antimicrobial peptides of multicellular organisms. Nature.

[2] Maroti G, Kereszt A, Kondorosi E, Mergaert P (2011). Natural roles of antimicrobial peptides in microbes, plants and animals. Res Microbiol.

[3] Bulet P, Stocklin R, Menin L (2004). Anti-microbial peptides: from invertebrates to vertebrates. Immunol Rev.

[4] Login FH (2011). Antimicrobial peptides keep insect endosymbionts under control. Science.

[5] Salzman NH (2009). Enteric defensins are essential regulators of intestinal microbial ecology. Nat Immunol.

[6] Tasiemski A (2015). Reciprocal immune benefit based on complementary production of antibiotics by the leech Hirudo verbana and its gut symbiont Aeromonas veronii. Sci Rep.

[7] Franzenburg S (2013). Distinct antimicrobial peptide expression determines host species-specific bacterial associations. Proc Natl Acad Sci USA.

[8] Gallo RL, Nakatsuji T (2011). Microbial symbiosis with the innate immune defense system of the skin. J Invest Dermatol.

[9] Tennessen JA (2005). Molecular evolution of animal antimicrobial peptides: widespread moderate positive selection. J Evol Biol.

[10] Gosset CC, Do Nascimento J, Auge MT, Bierne N (2014). Evidence for adaptation from standing genetic variation on an antimicrobial peptide gene in the mussel Mytilus edulis. Mol Ecol.

[11] Unckless RL, Howick VM, Lazzaro BP (2016). Convergent Balancing Selection on an Antimicrobial Peptide in Drosophila. Curr Biol.

[12] Unckless, R. L. & Lazzaro, B. P. The potential for adaptive maintenance of diversity in insect antimicrobial peptides. *Philos Trans R Soc Lond B Biol Sci* **371**, doi:10.1098/rstb.2015.0291 (2016). 10.1098/rstb.2015.0291 PMC4874389 27160594

[13] Salathé M, Kouyos RD, Bonhoeffer S (2008). The state of affairs in the kingdom of the Red Queen. Trends in Ecology & Evolution.

[14] Otti O, Tragust S, Feldhaar H (2014). Unifying external and internal immune defences. Trends Ecol Evol.

[15] Salzet M, Tasiemski A, Cooper E (2006). Innate immunity in lophotrochozoans: the annelids. Curr Pharm Des.

[16] Tasiemski A, Salzet M (2010). Leech immunity: from brain to peripheral responses. Adv Exp Med Biol.

[17] Conlon JM (2011). The contribution of skin antimicrobial peptides to the system of innate immunity in anurans. Cell Tissue Res.

[18] Schikorski D (2008). The medicinal leech as a model for studying the antimicrobial response of the central nervous system. J Immunol.

[19] Tasiemski A (2014). Characterization and function of the first antibiotic isolated from a vent organism: the extremophile metazoan Alvinella pompejana. PLoS ONE.

[20] Cary SC, Cottrell MT, Stein JL, Camacho F, Desbruyeres D (1997). Molecular Identification and Localization of Filamentous Symbiotic Bacteria Associated with the Hydrothermal Vent Annelid *Alvinella pompejana*. Appl Environ Microbiol.

[21] Le Bris N, Gaill F (2007). How does the annelid *Alvinella pompejana* deal with an extreme hydrothermal environment?. Rev Environ Sci Biotechnol.

[22] Bonch-Osmolovskaya EA (2011). Activity and distribution of thermophilic prokaryotes in hydrothermal fluid, sulfidic structures, and sheaths of alvinellids (East Pacific Rise, 13 degrees N). Appl Environ Microbiol.

[23] Di Meo-Savoie CA, Luther GW, Cary SC (2004). Physicochemical characterization of the microhabitat of the epibionts associated with *Alvinella pompejana*, a hydrothermal vent annelid. Geochim Cosmochim Acta.

[24] Luther GW (2001). Chemical speciation drives hydrothermal vent ecology. Nature.

[25] Le Bris N, Zbinden M, Gaill F (2005). Processes controlling the physico-chemical microenvironments associated with Pompeï worms. Deep-sea research.

[26] Cary SC, Shank T, Stein J (1998). Worms basks in extreme temperatures. Nature.

[27] Chevaldonné P, Desbruyères D, Le Haître M (1991). Time-series of temperature from three deep-sea hydrothermal vent sites. *Deep-Sea Res*. Part A.

[28] Grzymski JJ (2008). Metagenome analysis of an extreme microbial symbiosis reveals eurythermal adaptation and metabolic flexibility. Proc Natl Acad Sci USA.

[29] Doyle J, Doyle JL (1987). Genomic plant DNA preparation from fresh tissue-CTAB method. Phytochem. Bull.

[30] Jolly M, Viard F, Weinmayr G, Gentil F, Thiébaut E, Jollivet D (2003). Does the genetic the genetic structure of *Pectinaria koreni* (Polychaeta: Pectinariidae) conform to a source-sink metapopulation model at the scale of the Baie de Seine?. Helgoland Mar. Res..

[31] Bierne N (2007). Mark–recapture cloning: a straightforward and cost-effective cloning method for population genetics of single-copy nuclear DNA sequences in diploids. Molecular Ecology Notes.

[32] Heath L, van der Walt E, Varsani A, Martin DP (2006). Recombination patterns in aphthoviruses mirror those found in other picornaviruses. J. Virol..

[33] Librado P, Rozas J (2009). DnaSP v5: a software for comprehensive analysis of DNA polymorphism data. Bioinformatics.

[34] Fu YX (1997). Statistical tests of neutrality of mutations against population growth, hitchhiking and background selection. Genetics.

[35] Darriba D, Taboada GL, Doallo R, Posada D (2012). jModelTest 2: more models, new heuristics and parallel computing. Nat Meth.

[36] Tamura K, Stecher G, Peterson D, Filipski A, Kumar S (2013). MEGA6: Molecular Evolutionary Genetics Analysis Version 6.0. Molecular Biology and Evolution.

[37] Guindon S (2010). New Algorithms and Methods to Estimate Maximum-Likelihood Phylogenies: Assessing the Performance of PhyML 3.0. Systematic Biology.

[38] Tavaré S (1986). Some Probabilistic and Statistical Problems in the Analysis of DNA Sequences. American Mathematical Society: Lectures on Mathematics in the Life Sciences.

[39] Shoemaker JS, Fitch WM (1989). Evidence from nuclear sequences that invariable sites should be considered when sequence divergence is calculated. Molecular Biology and Evolution.

[40] Yang Z (1994). Maximum likelihood phylogenetic estimation from DNA sequences with variable rates over sites: Approximate methods. Journal of Molecular Evolution.

[41] Huson DH, Bryant D (2006). Application of phylogenetic networks in evolutionary studies. Mol Biol Evol.

[42] Yang Z (2007). PaML4: Phylogenetic analysis by maximum likelihood. Mol. Biol. Evol..

[43] Goldman N, Yang Z (1994). A codon-based model of nucleotide substitution for protein-coding DNA sequences. Mol Biol Evol.

[44] Thornton K, Long M (2005). Excess of Amino Acid Substitutions Relative to Polymorphism Between X-Linked Duplications in Drosophila melanogaster. Molecular Biology and Evolution.

[45] Arguello JR, Chen Y, Yang S, Wang W, Long M (2006). Origination of an X-Linked Testes Chimeric Gene by Illegitimate Recombination in Drosophila. PLOS Genetics.

[46] Meisel RP, Hilldorfer BB, Koch JL, Lockton S, Schaeffer SW (2010). Adaptive Evolution of Genes Duplicated from the Drosophila pseudoobscura neo-X Chromosome. Molecular Biology and Evolution.

[47] Hahn MW (2009). Distinguishing Among Evolutionary Models for the Maintenance of Gene Duplicates. Journal of Heredity.

[48] Ravaux J (2013). Thermal limit for metazoan life in question: *in vivo* heat tolerance of the pompeii worm. PLoS ONE.

[49] Schutte BC, Mitros JP, Bartlett JA (2002). Discovery of five conserved β-defensin gene clusters using a computational search strategy. Proceedings of the National Academy of Sciences.

[50] Semple, C. A. M., Rolfe, M. & Dorin, J. R. Duplication and selection in the evolution of primate b-defensin genes. *Genome Biol*. **4** (2003). 10.1186/gb-2003-4-5-r31 PMC156587 12734011

[51] Tennessen JA, Blouin MS (2007). Selection for antimicrobial peptide diversity in frogs leads to gene duplication and low allelic variation. J Mol Evol.

[52] Lynn DJ (2004). Bioinformatic discovery and initial characterisation of nine novel antimicrobial peptide genes in the chicken. Immunogenetics.

[53] Vrijenhoek, R. C. On the instability and evolutionary age of deep-sea chemosynthetic communities. *Deep Sea Res*. Part II, 189–200 (2013).

[54] Maxwell AI, Morrison GM, Dorin JR (2003). Rapid sequence divergence in mammalian beta-defensins by adaptive evolution. Mol Immunol.

[55] Elder JF, Turner BJ (1995). Concerted evolution of repetitive DNA sequences in eukaryotes. The Quarterly Review of Biology.

[56] Zhao M (2013). Evolution by selection, recombination, and gene duplication in MHC class I genes of two Rhacophoridae species. BMC Evol Biol.

[57] Awadalla P (2003). The evolutionary genomics of pathogen recombination. Nat Rev Genet.

[58] Fawcett JA, Innan H (2011). Neutral and non-neutral evolution of duplicated genes with gene conversion. Genes (Basel).

[59] Plouviez S, Le Guen D, Lecompte O, Lallier FH, Jollivet D (2010). Determining gene flow and the influence of selection across the equatorial barrier of the East Pacific Rise in the tube-dwelling polychaete Alvinella pompejana. BMC Evol Biol.

[60] Little CTS, Vrijenhoek RC (2003). Are hydrothermal vent animals living fossils?. Trends Ecol. Evol..

[61] Haymon RM, Koski RA, Sinclair C (1984). Fossils of hydrothermal vent worms from cretaceous sulfide ores of the samail ophiolite, oman. Science.

[62] Rolff, J. & Schmid-Hempel, P. Perspectives on the evolutionary ecology of arthropod antimicrobial peptides. *Philos Trans R Soc Lond B Biol Sci* **371**, doi:10.1098/rstb.2015.0297 (2016). 10.1098/rstb.2015.0297 PMC4874394 27160599

[63] Crovella S (2005). Primate beta-defensins–structure, function and evolution. Curr Protein Pept Sci.

[64] Lazzaro BP (2008). Natural selection on the Drosophila antimicrobial immune system. Curr Opin Microbiol.

[65] Cassone M, Otvos L (2010). Synergy among antibacterial peptides and between peptides and small-molecule antibiotics. Expert Rev Anti Infect Ther.

[66] Lauth X (2005). Bass hepcidin synthesis, solution structure, antimicrobial activities and synergism, and *in vivo* hepatic response to bacterial infections. J Biol Chem.

[67] Nagaoka I, Hirota S, Yomogida S, Ohwada A, Hirata M (2000). Synergistic actions of antibacterial neutrophil defensins and cathelicidins. Inflamm Res.

[68] Rosenfeld Y, Barra D, Simmaco M, Shai Y, Mangoni ML (2006). A synergism between temporins toward Gram-negative bacteria overcomes resistance imposed by the lipopolysaccharide protective layer. J Biol Chem.

[69] Yan H, Hancock RE (2001). Synergistic interactions between mammalian antimicrobial defense peptides. Antimicrob Agents Chemother.

[70] Knight SD, Presto J, Linse S, Johansson J (2013). The BRICHOS domain, amyloid fibril formation, and their relationship. Biochemistry.

[71] Sanchez-Pulido L, Devos D, Valencia A (2002). BRICHOS: a conserved domain in proteins associated with dementia, respiratory distress and cancer. Trends Biochem Sci.

[72] Willander H, Hermansson E, Johansson J, Presto J (2011). BRICHOS domain associated with lung fibrosis, dementia and cancer–a chaperone that prevents amyloid fibril formation?. FEBS J.

[73] Landreh M, Rising A, Presto J, Jornvall H, Johansson J (2015). Specific chaperones and regulatory domains in control of amyloid formation. J Biol Chem.

[74] Hedlund J, Johansson J, Persson B (2009). BRICHOS - a superfamily of multidomain proteins with diverse functions. BMC Res Notes.

[75] Jollivet D (2012). Proteome adaptation to high temperatures in the ectothermic hydrothermal vent Pompeii worm. PLoS One.

[76] James TC, Usher J, Campbell S, Bond U (2008). Lager yeasts possess dynamic genomes that undergo rearrangements and gene amplification in response to stress. Current Genetics.

