# Author Correction: Antagonistic evolution of an antibiotic and its molecular chaperone: how to maintain a vital ectosymbiosis in a highly fluctuating habitat

**DOI:** 10.1038/s41598-017-16211-w

**Published:** 2017-12-04

**Authors:** Claire Papot, François Massol, Didier Jollivet, Aurélie Tasiemski

**Affiliations:** 1 University Lille, CNRS, UMR 8198 - Evo-Eco-Paleo, SPICI group, F-59000 Lille, France; 2 AD2M, ABICE team, Université Pierre et Marie Curie-CNRS, UMR7144, Station Biologique de Roscoff, 29682 Roscoff, France

*Scientific Reports* **7**:1454; doi:10.1038/s41598-017-01626-2; Article published online 03 May 2017

Funding sources and the scientific contributions of B Shillito, J Ravaux and G Hamel were not acknowledged in this Article. The authors apologise for these accidental omissions. The Acknowledgements section,

“We thank the captain and crew of the RV Atalante, the DSV Nautile group (IFREMER), along with N. Le Bris (UMR8222) and F. Lallier (UMR7144), chief scientists of the MESCAL cruises. We thank A.S. Leport (UMR7144) for her technical assistance and B. Shillito (BOREA) and S. Hourdez (UMR7144) for the use of BALIST and DESEARES, respectively. We also acknowledge I. Probert (MBRC, Roscoff) for the English proofreading. This work was funded by the Université de Lille (BQR), the CNRS, the GDR ECCHIS and the Région Nord Pas de Calais-FRB (VERMER project)”.

should read:

“We thank the captain and crew of the RV Atalante, the DSV Nautile group (IFREMER), along with N. Le Bris (UMR8222) and F. Lallier (UMR7144), chief scientists of the MESCAL cruises. We thank A.S. Leport (UMR7144) for her technical assistance and B. Shillito (BOREA) and S. Hourdez (UMR7144) for the use of BALIST and DESEARES, respectively. We also acknowledge I. Probert (MBRC, Roscoff) for the English proofreading. This work was funded by the Université de Lille (BQR), the CNRS, the GDR ECCHIS and the Région Nord Pas de Calais-FRB (VERMER project). We thank B Shillito, J Ravaux (UMR 7208 BOREA) and G Hamel (UMR 7590 IMPMC), for conducting and performing the BALIST pressure experiments, and for providing access to the corresponding samples. The isobaric sampling and transfer equipments used in this study were funded by the EXOCET/D project (FP6 program, FP6-GOCE-CT-2003-505342), the BALIST project (ANR program, ANR-08-BLAN-0252), and by the University Pierre et Marie Curie (BQR)”.

In addition, the Author Contributions Statement is incorrect.

“C.P. generated and analyzed the sequence dataset. D.J. designed and contributed to the genetic analysis and performed the sampling with A.T. A.T. conducted and performed the experiments under pressure and the RT-qPCR. F.M. and C.P. participated in writing the paper. A.T. and D.J. supervised the work and wrote the paper”.

should read:

“C.P. generated and analyzed the sequence dataset. D.J. designed and contributed to the genetic analysis and performed the sampling with A.T. F.M. and C.P. participated in writing the paper. A.T. and D.J. supervised the work and wrote the paper”.

