# Supplementary material for: Antagonistic evolution of an antibiotic and its molecular chaperone: how to maintain a vital ectosymbiosis in a highly fluctuating habitat
Source: Sci Rep. 2017 May 3;7:1454. doi: 10.1038/s41598-017-01626-2 (PMC5431198; doi:10.1038/s41598-017-01626-2)
Supplement: Supplementary file 1 — SUPPLEMENTARY INFORMATION [file 41598_2017_1626_MOESM1_ESM.doc]

**Antagonistic evolution of an antibiotic and its molecular chaperone: how to maintain a vital ectosymbiosis in a highly fluctuating habitat**

Claire Papot, François Massol, Didier Jollivet, Aurélie Tasiemski

**SUPPLEMENTARY INFORMATION**

**EXTENDED EXPERIMENTAL PROCEDURES**

***Gene amplification.*** The preproalvinellacin gene displays a 5 introns/6 exons structure, with a first large intron of 449 bp after the signal peptide sequence ^1^ (Fig. 2). Because of its length, nested primers with a 100 bp overlap in the middle of the gene were designed to split the gene at the beginning of the BRICHOS domain (see table S1): the 5’ part comprises both the peptide signal and the linker region (3 exons + 3 introns) whereas the 3’ part contains the BRICHOS domain and the antimicrobial peptide (3 exons + 2 introns).

**Specimen sampling**

The genus *Alvinella* is made of only two species: the Pompeii worm *A. pompejana* and its closely related syntopic species *A. caudata*. Both species live on the wall of vent chimneys distributed along the East Pacific Rise (EPR). Animals were collected using the arm of the manned submersible Nautile and brought back to the surface inside an insulated basket. Until sampled, animals were measured, sexed and frozen immediately at -80°C for further laboratory analysis or alternatively freshly dissected to perform a DNA extraction directly onboard from the anterior part of the animal (gills and prostomium, which are devoid of epibionts). The two sampling sites are separated by a distance of about 3000km and a major faulting system (Quebrada/Discovery/Gofar fracture zone) around the Equator known to represent a biogeographic barrier for the vent fauna ^2^. *Alvinella* specimens from both sides of the EPR therefore display a *Cox-1* specific mitochondrial signature with 2% divergence.

**PCR amplification, cloning and sequencing**

Four *A. caudata* specimens and 96 *A. pompejana* were sampled from populations on both sides of the Equatorial barrier (50 individuals from Fromveur and 50 individuals from Bio9). Each individual was amplified by PCR separately with a different tag combination. PCR were conducted in a 25µL volume including: 1X buffer, 2mM MgCL2, 0.05mM of each dNTP, 0.4µM of each primer, 1U of Taq polymerase (Uptitherm^TM^, Interchim). Thermal cycling parameters used an initial denaturation step at 96°C for 4 min, followed by 40 cycles at 96°C for 30 s, 60°C for 45 s and 72°C for 2 min, before a 10 min final extension at 72°C. PCR products were then pooled together before cloning. In *A. pompejana*, the technique consisted in pooling the same amount of DNA from PCR amplification of 16 previously tagged individuals for each cloning assay and, to perform six distinct cloning assays for both the 5’ and 3’ regions of the gene. In other words, 12 pools were made representing 96 individuals for each part of the gene (i.e. 192 amplifications representing 384 allelic fragments for an autosomal locus). Pools of tagged PCR products were purified using QIAquick^TM^ columns and ligated into a Bluescript vector using the TOPO_TA cloning kit (Invitrogen^TM^) and subsequently transformed into top10 competent *E. coli* strain following the manufacturer’s instructions. For each cloning assay, after amplification of the insert with Puc-specific plasmid (outside the polyclonal region) BS1 primers, 96 positive clones (i.e. containing an insert of the right size) were sequenced on both strands with the sequencing primers M13F or M13R, leading to a total number of 1152 sequences and a recapture effort of 3.0 under the single locus assumption. The mark-recapture cloning method led to more than 900 proof-read sequences in both directions (321 in the 5’ region and 566 in the 3’ region) for the focus species *A. pompejana* and only 20 and 58 sequences in the 5’ and 3’ regions for the outgroup species *A. caudata*. A consensus sequence of the two forward and reverse reads was produced for each clone. Sequences recaptured more than once were the only sequences kept for the first allelic assignment to duplicated loci. The mark-recapture cloning technique generates about 30% of artifactual recombinants with our complex dataset when looking at the most recaptured individuals (c.a. > 20 clones). These chimeric alleles were either due to intra-locus or inter-loci recombination during the PCR. Some recombinants were, however, considered to be natural when recaptured in more than two individuals (i.e. alleles with the same recombination breakpoints in distinct individuals).

**Cleaning sequence datasets from artifactual mutational events**

Global alignments of consensus sequences were obtained with the Geneious software using ClustalW with the free ends gap option, a gap penalty of 1.0 and a cost matrix option of 51% similarity. Chimeras of alleles for heterozygous individuals or chimeras of alleles between closely-related loci in the specific case of duplicated genes were of frequent occurrences in our sequence datasets. Tracing back recombinants was, however, only possible for the most recaptured individuals displaying at least 20 clones mainly because of the high number of duplicated genes in our set of sequences. Hence, intra-individual *in vitro* recombination points between alleles and/or duplicated genes were searched using RDP4.0. First, for each set of clonal sequences attributed to one individual, the alignment-based “Automated MAxChi” procedure ^3^ was performed with all settings left as default. The within-individual alignment with ‘true’ allelic sequences and identified chimeras was thereafter checked manually to visualize any additional intra-individual recombinants. Second, putative recombinants in a given individual were compared with the whole sequence dataset to see whether they might be shared with other individuals. Recombinants found in more than one individual were kept and assumed to represent ‘natural’ intra- or inter-loci recombination events. Third, a second set of recombinant search was performed with the MAxChi procedure over the multi-individual alignment of the remaining sequences to identify additional recombining points across distinct individuals. These new recombinants were also removed from the dataset if not observe in at least two distinct individuals. This allowed us to confirm the natural existence of previously described alleles. Finally, alleles from individuals weakly recaptured or only recaptured once were added to the final dataset if they were able to match at least one sequence of the curated alignment. On this ‘cleaned’ dataset, artifactual/somatic mutations were also removed taking advantage of the multiple recaptures (i.e. >20 sequences) by suppressing singletons between intra-individual sequences that referred to a well-assigned allele. This allowed us to calculate a rate at which artifactual mutations occur in the dataset and to apply this rate on singletons found in the other less recaptured individuals.

**Paralog identification and individual genotyping**

Combining the 5’ and 3’ regions of the gene (separate PCR amplification) and thus, the exact correspondence of 5’ and 3’ alleles, was not possible in this study due to the high rate of recombination and the lack of diagnostic sites in the 100 bp-overlapping region of the gene, leading to a disjoint assignment of paralogs in the two genic regions. Exact allelic concordance was only met for 5’ paralog 5 and the 3’ paralog E as they both display the highest level of divergence with the other paralogs, respectively. A detailed analysis of paralogs was performed at the intraspecific level from the whole ‘cleaned’ sequence dataset recovered from *A. pompejana* on the 5’ region of the gene. This region was chosen because it contains long intronic regions that help to discriminate more easily between paralogs (i.e. specific signatures of linked sites). Forward and reverse paralog-specific primers were positioned on specific mutation signatures typifying each putative paralog with a final amplicon size of less than 400-nucleotides long. For each paralogous gene, direct sequencing allowed us to search for heterozygous individuals (double peaks in the chromatogram) at diagnostic sites using an alignment performed with the *de novo* Assemble module of the Geneious software. Gene orthology was confirmed for a given set of primers when both homozygous and heterozygous individuals co-occur at previously chosen diagnostic sites.

The evolutionary history of paralogs was inferred with the Maximum Likelihood method of the software MEGA6 using the GTR model of substitutions and the allelic alignment of either the 5’ or 3’ regions (coding and non-coding region) of the gene. Initial tree(s) for the heuristic search were obtained by applying the BioNJ method to a matrix of pairwise distances estimated using the Maximum Composite Likelihood (MCL) approach. A discrete Gamma distribution was used to model evolutionary rate differences among sites (4 categories (Gamma shape parameter = 0.13) with no invariable sites). The tree was drawn to scale with branch lengths measured as the number of substitutions per site. A search for the best model of substitutions was also performed using jModelTest 2.1.7. The tree topology obtained with the GTR+I+G model was compared with possible alternative trees. Results using the whole set of allelic sequences of the 5’ region of the gene indicated that the best substitution model is the GTR+G according to the AIC or the TPM3uf+G model according to the BIC, but the three models (GTR+I+G, GTR+G and TPM3uf+G) fall within the 95% confidence intervals of the AIC/BIC analyses (i.e. models which have a substantial support for the dataset by summing the ranked weight (ωi) of each model (i) that uses the difference between each model-specific value of AIC/BIC and the minimum one). There was no significant difference between the GTR+G and GTR+G+I models (LRT=2.22, df=1) and the TPM3uf model produced a significantly decreased likelihood than the GTR+G+I model (LRT=2.45, df=4). Comparing the topologies obtained with PhyML under the two selected best models and the GTR+G+I model did not give much difference in topology (see alternative topologies below). Slight differences in the coalescence of alleles within each paralog were observed but were not taken into account, as they have no influence on the arrangement of clades.

The phylogenetic network constructed via the NeighborNet method implemented in the program SplitsTree4 ^4^ indicated that the preproalvinellacin is encoded by a multigenic family of six genes (par1, par2, par3a, par3b, par4 and par5), some of the alleles being recently derived recombinants (Par5 R2) while others (Par5 R1, Par4-1 R) represent older recombinants that have already accumulated their proper set of mutations. Twelve unambiguous sequences were kept for par1, 6 for par2, 13 for par3a, 9 for par3b, 14 for par4 and 25 for par5. The length of alleles dramatically varied between paralogs, mainly because of indels in the intronic regions. No indel was depicted in the exonic regions with the exception of Par2 which lacks a piece of 34 codons located at the end of the first exon and the beginning of the second one without changing the reading frame. Par4 displayed the lowest length due to a major deletion in the first intronic region. Par5 was the most divergent lineage mainly because of the first intron, which exhibited a tandem repeat region and could not be aligned with the other sequences (foreign insertion due to an unequal crossing over with another gene).

**Strength of selection along the preproalvinellacin gene**

Intensity of selection acting on each domain of the gene (i.e. signal peptide, propiece, BRICHOS and AMP) according to each paralog was measured using the ratio of non-synonymous substitution rate (d_N_), which are usually subject to selective pressure, and the synonymous substitution rate (d_S_), which is assumed to be (nearly) neutral ^5,6^. Values greater than one were assumed to show positive diversifying selection on the divergence between two paralogous domains, and thus positive diversification of duplicates.

**Search for positive selection in the propiece and BRICHOS regions of preproalvinellacin** Paralogous consensus sequences of the propiece (79 sequences) and the BRICHOS plus the alvinellacin AMP (36 sequences) were aligned together using ClustalW of the alignment module of Geneious for *A. pompejana* and *A. caudata*. All positions with less than 95% site coverage were eliminated, leading to a total of 681 site positions in the final dataset. A search for the best model of substitutions was performed with jModelTest 2.1.7 and the tree topologies obtained from the PhyML reconstruction with these models were compared to the topology obtained with the GTR+I+G, as implemented in MEGA6 (Tables S2 and S3). Using the BRICHOS alignment, a hLRT backward selection procedure showed that none of the nested substitution models had significantly poorer goodness-of-fit than the GTR+I+G model (log likelihood=-439.625, BIC=1299.380) with the exception of the JC+I+G model (log likelihood= -452.175). Because the best model was the K80+I according to the BIC (log likelihood=-441.781, BIC=1261.15), we carefully examined the topology of the BIC-based best tree when compared to the GTR+I+G used for CodeML and aaML analyses (Fig. S3). Though more simplistic, the K80+I topology between the paralogous clades was not different from the one given by the GTR+I+G model and thus does not affect either the ancestral reconstruction or the search for positive selection on codons. For the propiece CodeML analysis (79 sequences), the AIC-based best model was also the K80+I model (log likelihood=-607.94, AICc= 3657.6) but the TPM2+I+G model (log likelihood=-588.29, BIC=1997.1) according to the BIC. Both models had better goodness-of-fit criteria than the GTR+I+G model (log likelihood=-579.8, AICc=5402.5, BIC=2016.5). However, hLRTs in backward selection indicated no significant differences in goodness-of-fit between nested models until the Tim2ef+I+G model. Tree reconstruction with this specific model did not modify the topology of the reference tree used for the Propiece analyses. Models H80+I and Tim2ef+I+G were then used for the tree reconstruction of a smaller set of duplicate-specific consensus sequences for either the BRICHOS or the Propiece domain (8 sequences each) and, subsequently used in the CodeML analyses for the article in order to remove polymorphic sequences from the analysis (Figs S3 and S4). Results from the small sets of consensus sequences are now provided in Table 2 and results from all sets of sequences (including polymorphic ones) are provided in Tables S6 and S7. Several nested models of codon selection (i.e. M_3_, M_2a_ and M_8_) were subsequently tested against their ‘nearly neutral’ counterparts (i.e. M_0_, M_1a_ and M_7_, respectively) using a likelihood ratio test (LRT). The codon sequence dataset was first fitted on the ‘nearly neutral’ model M_1a_, which divides codon sites into two categories, those under purifying selection (ω_0_<1) and the others under relaxed selection (ω_1_=1) using our reference tree. This model was then compared to the alternative nested ‘selection’ model M_2a_ where a third category of codon sites under positive selection (ω_2_>1) is added, thus accommodating positively selected sites. More sophisticated alternative nested models - the ‘nearly neutral beta’ M_7_ and the ‘selective beta’ M_8_ - were also compared. These models assume an omega distribution that follows a β (p,q) distribution with the shape parameter estimated in the interval [0, 1]. The difference between the two nested models lies in the fact that M_8_ includes one additional substitution rate ω_1_ with a probability p_1_ that accounts for positively selected sites. In these two models, the rate of synonymous substitutions (d_S_) is fixed among sites, while the rate of non-synonymous substitutions (d_N_) remained variable along the gene. The significance of selection models using a likelihood ratio test with a degree of freedom equal to the difference between the number of parameters estimated for the 2 models when comparing M_2A_ vs. M_1A_ and, M_8_ vs. M_7_ (adapted from ^7^).

**MacDonald-Kreitman test between pairs of paralogs**

Another approach to detect signs of positive selection, the MacDonald-Kreitman test, was also performed onto the propiece and BRICHOS regions of the *preproalvinellacin* taking advantage of the fixed divergence between paralogs. Under strict neutrality, both rates of synonymous and non-synonymous substitutions are expected to be equal in either the species divergence or the within-species polymorphisms, but d_N_/d_S_ would become much greater than p_N_/p_S_ under positive diversifying selection. None of the tests was significant but MK test can be easily biased if the constancy of the neutral accumulation of mutations is not met over time for duplicates ^8^. In order to test whether selective relaxation has occurred prior to the duplication events, we performed a Branch model analysis with CodeML by comparing the ‘free ratio’ model M_1_ to the ‘one ratio’ model M_0_ and checked whether the d_N_/d_S_ ratios were higher in the internal branches leading to the duplicates when compared to their associated terminal branches. For the BRICHOS domain, M_1_ (log likelihood=-370.654, np=29) was not significantly better (LRT=9.44, df=13, NS) than M_0_ (log likelihood=-375.374, np=16), with a nearly neutral evolution before and after duplication (overall omega=0.64). For the Propiece domain, M_1_ (log likelihood=-396.573, np=29) was also not significantly better (LRT=19.84, df=13, NS) than M_0_ (log likelihood=-406.491, np=16), but its evolution was even more relaxed with positive selection (overall omega=1.18). In this case, terminal branches (6 out of eight with omega>1 for the Propiece) produced much higher d_N_/d_S_ ratios than their internal counterparts when using the M_1_ branch-model.

**SUPPLEMENTARY FIGURES AND TABLES**

**Figure S1. BRICHOS and alvinellacin amino-acid alignment.** Sequence labels represent the individual and clone number and are representative of the 6 paralogous clades (excluding natural recombinants, see Fig. 2) subsequently used in the mapping of the BRICHOS mutations. Ac: *A. caudata*; Ap *A. pompejana*

AMP

PR


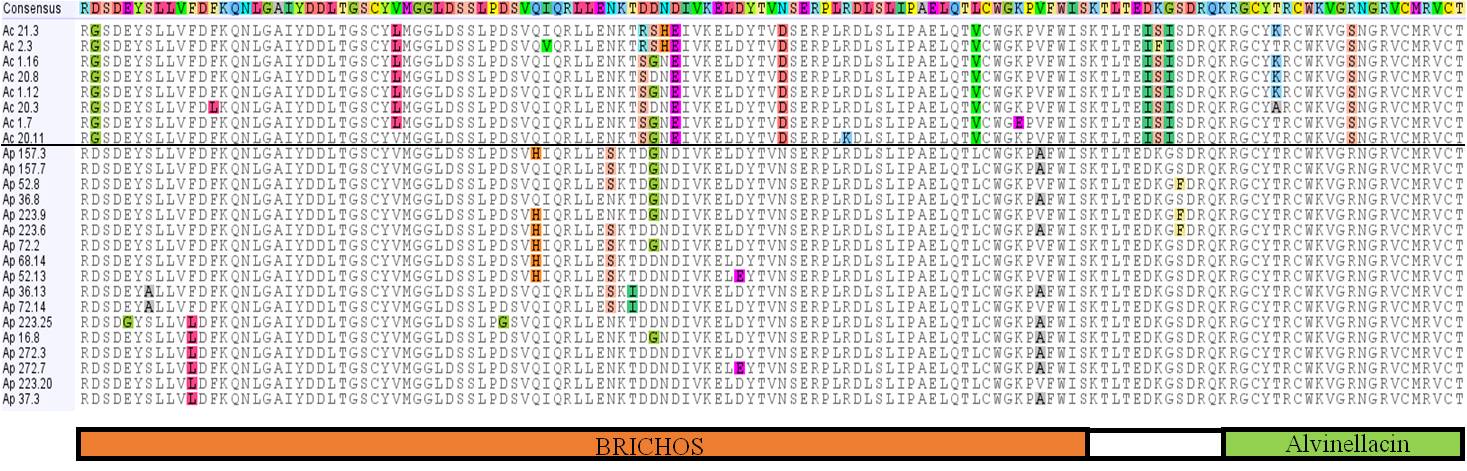


**Figure S2. Tree topology comparisons between GTR+I+G and the AIC- and BIC-based best models for the most variable 5’ region of the preproalvinellacin used in the identification of paralogous genes.** Comparison between topologies of the alvinellacin paralogous MEGA tree obtained in Figure 2 using (**A**) the GTR+I+G model implemented in MEGA 6.0 (log likelihood= 5367.044, AIC=14030.71, BIC=14981.24), (**B**) the selected best model (GTR+G) obtained with jModelTest v2.1.7 based on the AIC criterion (log likelihood=-5365.93, AIC=14025.04) and, (**C**) the selected best model (TPM3uf+G) obtained with jModelTest v2.1.7 based on the BIC criterion (log likelihood=-5368.27, BIC=14963.74). Note that these three models fall within the 95% confidence intervals of the jModelTest analysis, and that the three models are not significantly different according to hierarchical likelihood ratio tests (LRT). Slight differences can be observed in the gene genealogies of each paralogous clade but do not affect the clade rearrangement.


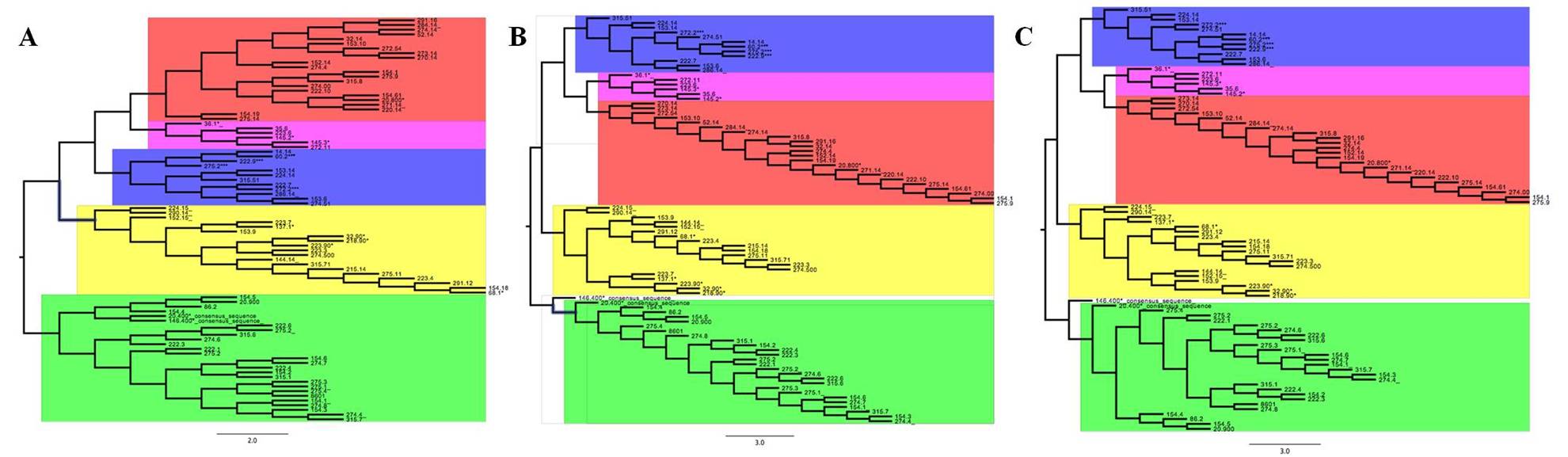


**Fig. S3. PhyML tree of the BRICHOS domain using the K80+I model selected by jmodeltest according to the BIC.** Topology of BRICHOS trees obtained using the K80+I model selected by jModelTest 2.1.7 according to the BIC criterion (log likelihood=-441.781, BIC=1261.15). This tree topology was not different from the one obtained using the GTR+I+G model implemented in MEGA 6.0 (Fig. 4 in main text) and led to the exact same conclusions when used as the reference tree in the CodeML and aaML analyses.


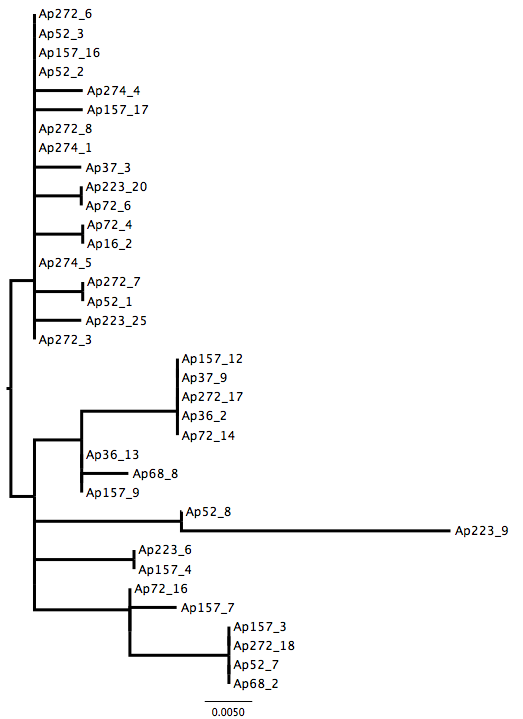


**Fig. S4. Propiece ML tree topology using the GTR+I+G model implemented in MEGA 6.0 and the BIC-based best model (TPM2ef+I+G) obtained from the jModelTest analysis.** Both models led to the same topology and all the 88 substitution models tested fall within the 95% confidence interval of the AIC/BIC analyses (see text). (**A**) Ultrametric and (**B**) chronogram representations of the same tree topology.


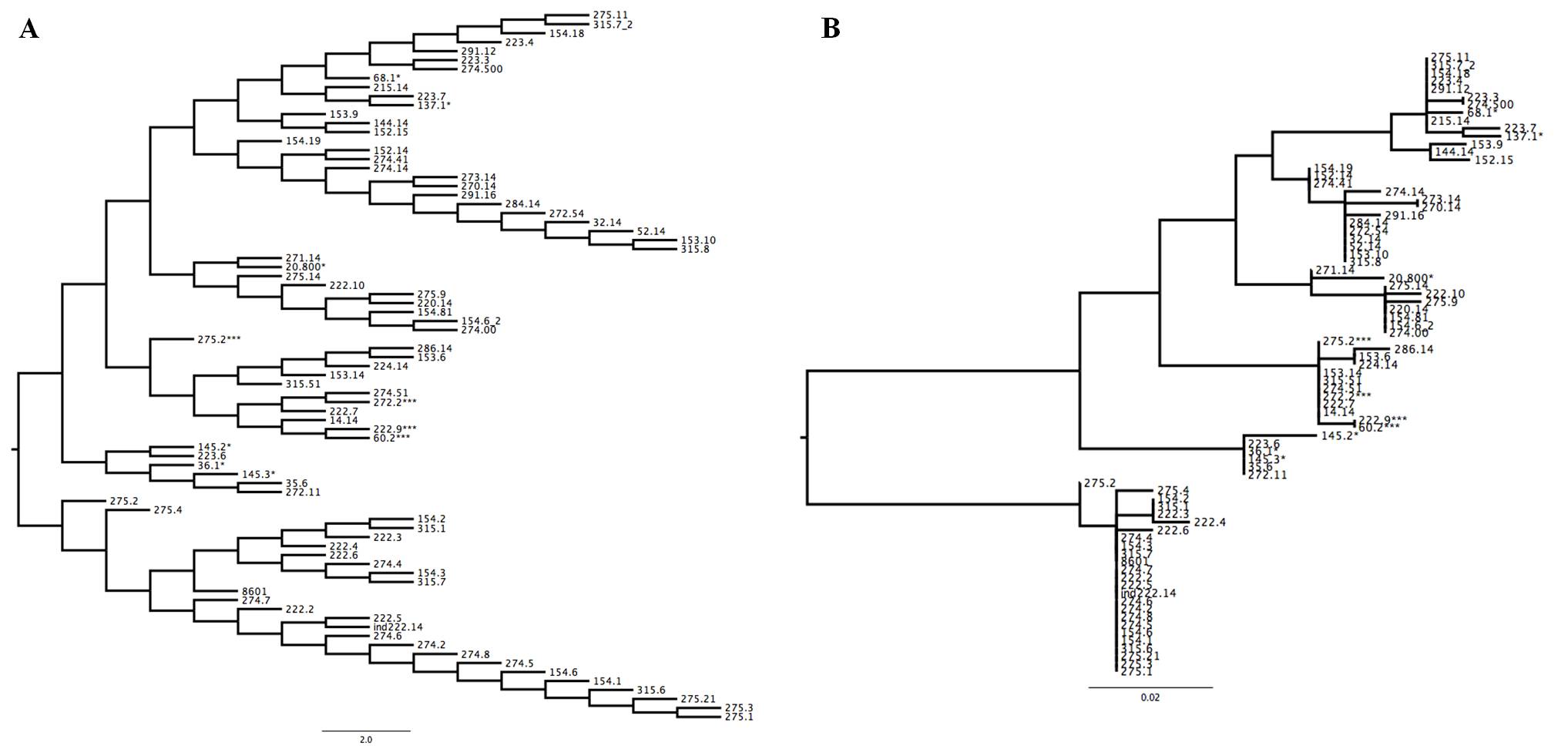


**Table S1**. Primers sequences (Forward and Reverse) used for the amplification of the preproalvinellacin gene from both *Alvinella pompejana* and *Alvinella caudata* and used for the genotyping of each paralogs (Px) for the *Alvinella pompejana* species.

| *Alvinella pompejana* |  |  |
| --- | --- | --- |
|  | 3' Forward | ATCGTGTTACGTCATGGGTGGCCTTG |
|  | 3' Reverse | CTCAGTGAAATGAAGCAGGTGAGTTATG |
|  | 5' Forward | ATGACGTATTCTGTAGTTGTGACGCTGGTC |
|  | 5' Reverse | ATCCGGTAAGATCGTCGTAAATGGCTCC |
| Genotyping |  |  |
|  | P1_Forward | ACATCTACAGATTGGTGCTATCGAC |
|  | P2_Forward | CTACAGATTGGTGCAGCCGAC |
|  | P3_Forward | CATCTACAGATTGGTGCTGTGGAT |
|  | P4_Forward | AACAGATTGGTGCTGTCGCC |
|  | P5_Forward | TTTACATAGATTGGTGTTTCTTCTCTGAG |
|  | P1_Reverse | GTTGAGGTGGCCAGCTGC |
|  | P2-Reverse | GTTGGGGTGGCCAACTGC |
|  | P3_Reverse | ATGTTGGGGTGATCAGCTGC |
|  | P4_Reverse | GATGTTGAGGTGGCCAGCTAT |
|  | P5_Reverse | GTTTCATGAAATGTGGCAGATG |
| *Alvinella caudata* |  |  |
|  | 5' Forward | GTTACGTATTCTGTAGTCACGACGCTG |
|  | 5' Reverse | GGTAAGATCGTCGTAAATGGCTCC |
|  | 3' Forward | GTCGTGTTACCTGATGGGTGGC |
|  | 3' Reverse | AATATGCCAAAACAGGCGAATTACG |

**Table S2. AIC-based goodness-of-fit indicators of substitution models for the most variable 5’ region of the preproalvinellacin used in the identification of paralogous genes, obtained from jModelTest.** Models are ordered by increasing AIC.

| **Model** | **- log likelihood** | **number of parameters** | **AIC** | **ΔAIC** | **Akaike weight** | **Cumulative weight** |
| --- | --- | --- | --- | --- | --- | --- |
| GTR+G | 6833.52 | 179 | 14025.04 | 0.00 | 0.75 | 0.75 |
| TVM+G | 6836.38 | 178 | 14028.75 | 3.71 | 0.12 | 0.87 |
| GTR+I+G | 6835.36 | 180 | 14030.71 | 5.67 | 0.04 | 0.92 |
| TIM3+G | 6838.39 | 177 | 14030.79 | 5.75 | 0.04 | 0.96 |
| TVM+I+G | 6836.73 | 179 | 14031.46 | 6.42 | 0.03 | 0.99 |
| TPM3uf+G | 6841.17 | 176 | 14034.33 | 9.29 | 0.01 | 1.00 |
| TIM3+I+G | 6840.53 | 178 | 14037.07 | 12.03 | 0.00 | 1.00 |
| TPM3uf+I+G | 6841.72 | 177 | 14037.43 | 12.39 | 0.00 | 1.00 |
| TIM2+G | 6842.97 | 177 | 14039.95 | 14.91 | 0.00 | 1.00 |
| TPM2uf+G | 6844.39 | 176 | 14040.79 | 15.75 | 0.00 | 1.00 |
| TrN+G | 6847.11 | 176 | 14046.22 | 21.18 | 0.00 | 1.00 |
| TIM2+I+G | 6845.54 | 178 | 14047.08 | 22.04 | 0.00 | 1.00 |
| TPM2uf+I+G | 6847.15 | 177 | 14048.29 | 23.25 | 0.00 | 1.00 |
| HKY+G | 6850.36 | 175 | 14050.72 | 25.68 | 0.00 | 1.00 |
| TIM1+G | 6848.95 | 177 | 14051.90 | 26.86 | 0.00 | 1.00 |
| TPM1uf+G | 6850.07 | 176 | 14052.15 | 27.11 | 0.00 | 1.00 |
| TrN+I+G | 6849.80 | 177 | 14053.60 | 28.56 | 0.00 | 1.00 |
| GTR+I | 6848.00 | 179 | 14054.00 | 28.96 | 0.00 | 1.00 |
| HKY+I+G | 6851.19 | 176 | 14054.38 | 29.34 | 0.00 | 1.00 |
| TIM1+I+G | 6849.49 | 178 | 14054.97 | 29.93 | 0.00 | 1.00 |
| TVM+I | 6849.67 | 178 | 14055.35 | 30.31 | 0.00 | 1.00 |
| TPM1uf+I+G | 6850.88 | 177 | 14055.76 | 30.72 | 0.00 | 1.00 |
| TIM3+I | 6853.24 | 177 | 14060.49 | 35.45 | 0.00 | 1.00 |
| TPM3uf+I | 6854.83 | 176 | 14061.66 | 36.63 | 0.00 | 1.00 |
| TIM2+I | 6858.26 | 177 | 14070.53 | 45.49 | 0.00 | 1.00 |
| TPM2uf+I | 6859.96 | 176 | 14071.93 | 46.89 | 0.00 | 1.00 |
| TrN+I | 6862.51 | 176 | 14077.01 | 51.97 | 0.00 | 1.00 |
| HKY+I | 6864.07 | 175 | 14078.14 | 53.10 | 0.00 | 1.00 |
| TIM1+I | 6862.28 | 177 | 14078.57 | 53.53 | 0.00 | 1.00 |
| TPM1uf+I | 6863.76 | 176 | 14079.51 | 54.47 | 0.00 | 1.00 |
| TPM3+I+G | 6870.38 | 174 | 14088.76 | 63.72 | 0.00 | 1.00 |
| TIM3ef+I+G | 6869.91 | 175 | 14089.82 | 64.78 | 0.00 | 1.00 |
| TVMef+I+G | 6869.05 | 176 | 14090.10 | 65.06 | 0.00 | 1.00 |
| SYM+I+G | 6868.59 | 177 | 14091.18 | 66.14 | 0.00 | 1.00 |
| TPM3+G | 6873.36 | 173 | 14092.73 | 67.69 | 0.00 | 1.00 |
| K80+I+G | 6873.63 | 173 | 14093.25 | 68.21 | 0.00 | 1.00 |
| TPM1+I+G | 6872.68 | 174 | 14093.36 | 68.32 | 0.00 | 1.00 |
| TIM3ef+G | 6872.94 | 174 | 14093.88 | 68.84 | 0.00 | 1.00 |
| TIM1ef+I+G | 6872.21 | 175 | 14094.42 | 69.38 | 0.00 | 1.00 |
| TVMef+G | 6872.27 | 175 | 14094.54 | 69.50 | 0.00 | 1.00 |
| TrNef+I+G | 6873.27 | 174 | 14094.55 | 69.51 | 0.00 | 1.00 |
| TPM2+I+G | 6873.52 | 174 | 14095.05 | 70.01 | 0.00 | 1.00 |
| SYM+G | 6871.85 | 176 | 14095.69 | 70.65 | 0.00 | 1.00 |
| TIM2ef+I+G | 6873.06 | 175 | 14096.12 | 71.08 | 0.00 | 1.00 |
| K80+G | 6876.61 | 172 | 14097.22 | 72.18 | 0.00 | 1.00 |
| TPM1+G | 6875.85 | 173 | 14097.70 | 72.66 | 0.00 | 1.00 |
| TrNef+G | 6876.18 | 173 | 14098.37 | 73.33 | 0.00 | 1.00 |
| TIM1ef+G | 6875.42 | 174 | 14098.84 | 73.80 | 0.00 | 1.00 |
| TPM2+G | 6876.54 | 173 | 14099.08 | 74.04 | 0.00 | 1.00 |
| TIM2ef+G | 6876.12 | 174 | 14100.24 | 75.20 | 0.00 | 1.00 |
| TPM3+I | 6889.00 | 173 | 14123.99 | 98.95 | 0.00 | 1.00 |
| TIM3ef+I | 6888.42 | 174 | 14124.83 | 99.79 | 0.00 | 1.00 |
| TVMef+I | 6887.70 | 175 | 14125.40 | 100.36 | 0.00 | 1.00 |
| SYM+I | 6887.12 | 176 | 14126.24 | 101.20 | 0.00 | 1.00 |
| K80+I | 6892.58 | 172 | 14129.16 | 104.12 | 0.00 | 1.00 |
| TPM1+I | 6891.71 | 173 | 14129.42 | 104.38 | 0.00 | 1.00 |
| TIM1ef+I | 6891.14 | 174 | 14130.29 | 105.25 | 0.00 | 1.00 |
| TrNef+I | 6892.23 | 173 | 14130.45 | 105.41 | 0.00 | 1.00 |
| TPM2+I | 6892.44 | 173 | 14130.89 | 105.85 | 0.00 | 1.00 |
| TIM2ef+I | 6891.88 | 174 | 14131.77 | 106.73 | 0.00 | 1.00 |
| F81+G | 6931.81 | 174 | 14211.62 | 186.58 | 0.00 | 1.00 |
| F81+I+G | 6932.11 | 175 | 14214.22 | 189.18 | 0.00 | 1.00 |
| F81+I | 6945.08 | 174 | 14238.17 | 213.13 | 0.00 | 1.00 |
| TVM | 6943.77 | 177 | 14241.53 | 216.49 | 0.00 | 1.00 |
| GTR | 6943.17 | 178 | 14242.33 | 217.29 | 0.00 | 1.00 |
| TPM3uf | 6948.84 | 175 | 14247.69 | 222.65 | 0.00 | 1.00 |
| TIM3 | 6948.29 | 176 | 14248.57 | 223.53 | 0.00 | 1.00 |
| JC+G | 6954.45 | 171 | 14250.90 | 225.86 | 0.00 | 1.00 |
| JC+I+G | 6955.38 | 172 | 14254.76 | 229.73 | 0.00 | 1.00 |
| TPM2uf | 6956.76 | 175 | 14263.52 | 238.48 | 0.00 | 1.00 |
| TIM2 | 6956.10 | 176 | 14264.19 | 239.15 | 0.00 | 1.00 |
| HKY | 6960.49 | 174 | 14268.98 | 243.94 | 0.00 | 1.00 |
| TrN | 6959.87 | 175 | 14269.75 | 244.71 | 0.00 | 1.00 |
| TPM1uf | 6960.14 | 175 | 14270.28 | 245.24 | 0.00 | 1.00 |
| TIM1 | 6959.54 | 176 | 14271.08 | 246.04 | 0.00 | 1.00 |
| JC+I | 6970.40 | 171 | 14282.79 | 257.75 | 0.00 | 1.00 |
| TPM3 | 6987.80 | 172 | 14319.61 | 294.57 | 0.00 | 1.00 |
| TVMef | 6985.82 | 174 | 14319.64 | 294.60 | 0.00 | 1.00 |
| TIM3ef | 6987.64 | 173 | 14321.27 | 296.23 | 0.00 | 1.00 |
| SYM | 6985.65 | 175 | 14321.31 | 296.27 | 0.00 | 1.00 |
| TPM1 | 6991.78 | 172 | 14327.57 | 302.53 | 0.00 | 1.00 |
| K80 | 6993.01 | 171 | 14328.01 | 302.97 | 0.00 | 1.00 |
| TIM1ef | 6991.59 | 173 | 14329.18 | 304.14 | 0.00 | 1.00 |
| TPM2 | 6992.77 | 172 | 14329.54 | 304.50 | 0.00 | 1.00 |
| TrNef | 6992.87 | 172 | 14329.74 | 304.70 | 0.00 | 1.00 |
| TIM2ef | 6992.58 | 173 | 14331.15 | 306.11 | 0.00 | 1.00 |
| F81 | 7034.17 | 173 | 14414.33 | 389.29 | 0.00 | 1.00 |
| JC | 7061.49 | 170 | 14462.99 | 437.95 | 0.00 | 1.00 |

**Table S3. BIC-based goodness-of-fit indicators of substitution models for the most variable 5’ region of the preproalvinellacin used in the identification of paralogous genes, obtained from jModelTest.** Models are ordered by increasing BIC.

| **Model** | **- log likelihood** | **number of parameters** | **AIC** | **ΔAIC** | **Akaike weight** | **Cumulative weight** |
| --- | --- | --- | --- | --- | --- | --- |
| TPM3uf+G | 6841.17 | 176 | 14963.74 | 0.00 | 0.62 | 0.62 |
| TIM3+G | 6838.39 | 177 | 14965.47 | 1.73 | 0.26 | 0.88 |
| TVM+G | 6836.38 | 178 | 14968.72 | 4.98 | 0.05 | 0.93 |
| TPM2uf+G | 6844.39 | 176 | 14970.19 | 6.45 | 0.02 | 0.96 |
| GTR+G | 6833.52 | 179 | 14970.28 | 6.55 | 0.02 | 0.98 |
| TPM3uf+I+G | 6841.72 | 177 | 14972.12 | 8.38 | 0.01 | 0.99 |
| TIM2+G | 6842.97 | 177 | 14974.63 | 10.89 | 0.00 | 0.99 |
| HKY+G | 6850.36 | 175 | 14974.84 | 11.11 | 0.00 | 1.00 |
| TrN+G | 6847.11 | 176 | 14975.62 | 11.89 | 0.00 | 1.00 |
| TVM+I+G | 6836.73 | 179 | 14976.70 | 12.97 | 0.00 | 1.00 |
| TIM3+I+G | 6840.53 | 178 | 14977.03 | 13.30 | 0.00 | 1.00 |
| GTR+I+G | 6835.36 | 180 | 14981.24 | 17.50 | 0.00 | 1.00 |
| TPM1uf+G | 6850.07 | 176 | 14981.55 | 17.81 | 0.00 | 1.00 |
| TPM2uf+I+G | 6847.15 | 177 | 14982.98 | 19.24 | 0.00 | 1.00 |
| HKY+I+G | 6851.19 | 176 | 14983.79 | 20.05 | 0.00 | 1.00 |
| TIM1+G | 6848.95 | 177 | 14986.58 | 22.84 | 0.00 | 1.00 |
| TIM2+I+G | 6845.54 | 178 | 14987.04 | 23.31 | 0.00 | 1.00 |
| TrN+I+G | 6849.80 | 177 | 14988.28 | 24.55 | 0.00 | 1.00 |
| TPM1uf+I+G | 6850.88 | 177 | 14990.44 | 26.70 | 0.00 | 1.00 |
| TPM3uf+I | 6854.83 | 176 | 14991.07 | 27.33 | 0.00 | 1.00 |
| TIM1+I+G | 6849.49 | 178 | 14994.94 | 31.20 | 0.00 | 1.00 |
| TIM3+I | 6853.24 | 177 | 14995.17 | 31.43 | 0.00 | 1.00 |
| TVM+I | 6849.67 | 178 | 14995.31 | 31.57 | 0.00 | 1.00 |
| GTR+I | 6848.00 | 179 | 14999.25 | 35.51 | 0.00 | 1.00 |
| TPM2uf+I | 6859.96 | 176 | 15001.33 | 37.59 | 0.00 | 1.00 |
| HKY+I | 6864.07 | 175 | 15002.26 | 38.52 | 0.00 | 1.00 |
| TIM2+I | 6858.26 | 177 | 15005.21 | 41.47 | 0.00 | 1.00 |
| K80+G | 6876.61 | 172 | 15005.50 | 41.76 | 0.00 | 1.00 |
| TPM3+G | 6873.36 | 173 | 15006.29 | 42.55 | 0.00 | 1.00 |
| TrN+I | 6862.51 | 176 | 15006.41 | 42.68 | 0.00 | 1.00 |
| K80+I+G | 6873.63 | 173 | 15006.81 | 43.08 | 0.00 | 1.00 |
| TPM3+I+G | 6870.38 | 174 | 15007.60 | 43.86 | 0.00 | 1.00 |
| TPM1uf+I | 6863.76 | 176 | 15008.91 | 45.18 | 0.00 | 1.00 |
| TPM1+G | 6875.85 | 173 | 15011.26 | 47.52 | 0.00 | 1.00 |
| TrNef+G | 6876.18 | 173 | 15011.93 | 48.19 | 0.00 | 1.00 |
| TPM1+I+G | 6872.68 | 174 | 15012.20 | 48.47 | 0.00 | 1.00 |
| TPM2+G | 6876.54 | 173 | 15012.64 | 48.91 | 0.00 | 1.00 |
| TIM3ef+G | 6872.94 | 174 | 15012.72 | 48.98 | 0.00 | 1.00 |
| TIM1+I | 6862.28 | 177 | 15013.25 | 49.52 | 0.00 | 1.00 |
| TrNef+I+G | 6873.27 | 174 | 15013.39 | 49.65 | 0.00 | 1.00 |
| TPM2+I+G | 6873.52 | 174 | 15013.89 | 50.15 | 0.00 | 1.00 |
| TIM3ef+I+G | 6869.91 | 175 | 15013.95 | 50.21 | 0.00 | 1.00 |
| TIM1ef+G | 6875.42 | 174 | 15017.68 | 53.95 | 0.00 | 1.00 |
| TIM1ef+I+G | 6872.21 | 175 | 15018.54 | 54.80 | 0.00 | 1.00 |
| TVMef+G | 6872.27 | 175 | 15018.66 | 54.92 | 0.00 | 1.00 |
| TIM2ef+G | 6876.12 | 174 | 15019.08 | 55.34 | 0.00 | 1.00 |
| TVMef+I+G | 6869.05 | 176 | 15019.50 | 55.77 | 0.00 | 1.00 |
| TIM2ef+I+G | 6873.06 | 175 | 15020.24 | 56.51 | 0.00 | 1.00 |
| SYM+G | 6871.85 | 176 | 15025.10 | 61.36 | 0.00 | 1.00 |
| SYM+I+G | 6868.59 | 177 | 15025.86 | 62.12 | 0.00 | 1.00 |
| K80+I | 6892.58 | 172 | 15037.44 | 73.70 | 0.00 | 1.00 |
| TPM3+I | 6889.00 | 173 | 15037.55 | 73.82 | 0.00 | 1.00 |
| TPM1+I | 6891.71 | 173 | 15042.98 | 79.24 | 0.00 | 1.00 |
| TIM3ef+I | 6888.42 | 174 | 15043.67 | 79.94 | 0.00 | 1.00 |
| TrNef+I | 6892.23 | 173 | 15044.01 | 80.28 | 0.00 | 1.00 |
| TPM2+I | 6892.44 | 173 | 15044.45 | 80.71 | 0.00 | 1.00 |
| TIM1ef+I | 6891.14 | 174 | 15049.13 | 85.39 | 0.00 | 1.00 |
| TVMef+I | 6887.70 | 175 | 15049.52 | 85.79 | 0.00 | 1.00 |
| TIM2ef+I | 6891.88 | 174 | 15050.61 | 86.87 | 0.00 | 1.00 |
| SYM+I | 6887.12 | 176 | 15055.64 | 91.90 | 0.00 | 1.00 |
| F81+G | 6931.81 | 174 | 15130.46 | 166.73 | 0.00 | 1.00 |
| F81+I+G | 6932.11 | 175 | 15138.34 | 174.60 | 0.00 | 1.00 |
| JC+G | 6954.45 | 171 | 15153.90 | 190.16 | 0.00 | 1.00 |
| F81+I | 6945.08 | 174 | 15157.01 | 193.27 | 0.00 | 1.00 |
| JC+I+G | 6955.38 | 172 | 15163.04 | 199.31 | 0.00 | 1.00 |
| TPM3uf | 6948.84 | 175 | 15171.81 | 208.07 | 0.00 | 1.00 |
| TVM | 6943.77 | 177 | 15176.21 | 212.48 | 0.00 | 1.00 |
| TIM3 | 6948.29 | 176 | 15177.98 | 214.24 | 0.00 | 1.00 |
| GTR | 6943.17 | 178 | 15182.30 | 218.56 | 0.00 | 1.00 |
| JC+I | 6970.40 | 171 | 15185.79 | 222.05 | 0.00 | 1.00 |
| TPM2uf | 6956.76 | 175 | 15187.64 | 223.90 | 0.00 | 1.00 |
| HKY | 6960.49 | 174 | 15187.82 | 224.09 | 0.00 | 1.00 |
| TIM2 | 6956.10 | 176 | 15193.60 | 229.86 | 0.00 | 1.00 |
| TrN | 6959.87 | 175 | 15193.87 | 230.13 | 0.00 | 1.00 |
| TPM1uf | 6960.14 | 175 | 15194.40 | 230.66 | 0.00 | 1.00 |
| TIM1 | 6959.54 | 176 | 15200.48 | 236.75 | 0.00 | 1.00 |
| TPM3 | 6987.80 | 172 | 15227.89 | 264.15 | 0.00 | 1.00 |
| K80 | 6993.01 | 171 | 15231.01 | 267.27 | 0.00 | 1.00 |
| TIM3ef | 6987.64 | 173 | 15234.83 | 271.10 | 0.00 | 1.00 |
| TPM1 | 6991.78 | 172 | 15235.85 | 272.11 | 0.00 | 1.00 |
| TPM2 | 6992.77 | 172 | 15237.82 | 274.08 | 0.00 | 1.00 |
| TrNef | 6992.87 | 172 | 15238.02 | 274.29 | 0.00 | 1.00 |
| TVMef | 6985.82 | 174 | 15238.48 | 274.74 | 0.00 | 1.00 |
| TIM1ef | 6991.59 | 173 | 15242.74 | 279.01 | 0.00 | 1.00 |
| TIM2ef | 6992.58 | 173 | 15244.71 | 280.98 | 0.00 | 1.00 |
| SYM | 6985.65 | 175 | 15245.43 | 281.69 | 0.00 | 1.00 |
| F81 | 7034.17 | 173 | 15327.89 | 364.16 | 0.00 | 1.00 |
| JC | 7061.49 | 170 | 15360.71 | 396.97 | 0.00 | 1.00 |

**Table S4. D_xy_ between paralogs**

|  | par5 | par1 | par2 | par3a | par3b | par4 |
| --- | --- | --- | --- | --- | --- | --- |
| par5 |  |  |  |  |  |  |
| par1 | 0.306 |  |  |  |  |  |
| par2 | 0.374 | 0.0921 |  |  |  |  |
| par3a | 0.307 | 0.0752 | 0.122 |  |  |  |
| par3b | 0.304 | 0.0769 | 0.111 | 0.0169 |  |  |
| par4 | 0.281 | 0.046 | 0.0987 | 0.0759 | 0.07789 |  |

**Table S5.** **Pairwise comparisons of K_a_/K_s_ ratios between paralogs (par) for each domain of the gene.** SP: signal peptide; PR: propiece; BRICHOS: BRICHOS domain, AMP: Antimicrobial Peptide mature domain. M refers to monomorphic sequences: no genetic diversity can be depicted for the pairwise comparison.

| **Region** | **Domain** | **Paralog** |  |  | **Paralog** |  |  |  |
| --- | --- | --- | --- | --- | --- | --- | --- | --- |
|  |  |  | par5 | par1 | par2 | par3a | par3b | par4 |
|  |  | par5 |  |  |  |  |  |  |
|  |  | par1 | 1.5842 |  |  |  |  |  |
|  | **SP** | par2 | 0.3786 | 0.8005 |  |  |  |  |
|  |  | par3a | 0.3597 | 0.7612 | 0 |  |  |  |
|  |  | par3b | 0.3597 | 0.7612 | 0 | 0 |  |  |
| 5’ |  | par4 | 0.7772 | 0.7612 | 0 | 0 | 0 |  |
|  |  |  | par5 | par1 | par2 | par3a | par3b | par4 |
|  |  | par5 |  |  |  |  |  |  |
|  |  | par1 | 1.5667 |  |  |  |  |  |
|  | **PR** | par2 | 1.9158 | 0.858 |  |  |  |  |
|  |  | par3a | 1.3084 | 0.2798 | 0.3757 |  |  |  |
|  |  | par3b | 1.0855 | 0.3998 | 0.4868 | 1.4101 |  |  |
|  |  | par4 | 0.9976 | 0.327 | 0.492 | 0.252 | 0.5152 |  |
|  |  |  | parE | parA | parB | parC | parD |  |
|  |  | parE |  |  |  |  |  |  |
|  | **BRICHOS** | parA | 0.5741 |  |  |  |  |  |
|  |  | parB | 1.8245 | 0.214 |  |  |  |  |
|  |  | parC | 0.5629 | 0.4216 | 0.8065 |  |  |  |
| 3’ |  | parD | 0.8603 | 0.8649 | 1.8177 | 0.2782 |  |  |
|  |  |  | parE | parA | parB | parC | parD |  |
|  |  | parE |  |  |  |  |  |  |
|  | **AMP** | parA | M |  |  |  |  |  |
|  |  | parB | M | M |  |  |  |  |
|  |  | parC | M | M | M |  |  |  |
|  |  | parD | M | M | M | M |  |  |

**Table S6.** **Log-likelihood values and parameter estimates for the BRICHOS domain region and the propiece of the preproalvinellacin gene.** Maximum-likelihood models implemented in the codeML program of the PAML package for models that allow positive selection (M2, M3,M8) and those that do not (M0, M1, M7). M0, one-ratio; M1, neutral; M2, selection; M3, discrete; M7, β; M8, β+ω and the estimated log-likelihood values (l) by the codeml program, ω = dN/dS nonsynonymous/synonymous rate ratio; p = proportion of sites for each site class. M0: one estimated ω for all sites; M1a: estimate p_0_= proportion of sites with ω_0_= 0,p_1_= 1 - p_0_, proportion of sites with ω_1_= 1; M2a: estimate p_0_(ω_0_= 0), p_1_(ω_0_= 1), and ω_2_, p_2_= 1 - p_0_- p_1_. M3: estimate p_0_, p_1_, ω_0_, ω_1_, and ω_2_; p_2_= 1 - p_0_- p_1_. M7: estimates p and q (parameters of β distribution of ω between 0 and 1). M8: same as M7 except additional site class where an estimated ω is allowed.  **Positively Selected Sites**: Codon positions predicted to be under positive selection with a posterior probability of ** >0.99 and *>95% (identification of sites exhibiting dn/dS ratio >1). Sites refer to amino acids positions from the first M. *: P>95%; **: P>99 in the Naive Empirical Bayes (NEB) analyses of PAML.

| **Model** |  | M0 | M3 (Discrete) | M1 (Neutral) | M2 (Selection) | M7 (β) | M8 (β+ω) |
| --- | --- | --- | --- | --- | --- | --- | --- |
| **BRICHOS** | **Log likelihood** | -443.76 | -437.09 | -439.14 | -437.09 | -439.17 | -437.09 |
|  | **Parameters estimates** | ω= 0,612 | ω0=0, p0=0.79, ω1=2.78772 p1=0.18, ω2=2.78775 p2=0.03 | ω0= 0, p0=0.67, (ω1=1) p1=0.33 | ω0=0, p0=0.79, ω1=1 p1=0, ω2=2.78 p2=0.21 | p=0.005, q=0.0117 | p0=0.78, p=0.005, p1=0.21, q= 2.74, ω=2.78 |
|  | **Sites with dN/dS>1**  **(NEB analysis)** | n.a. | D119G; Q121H; N129S; T131I; D133G; D141E; V169A (all **) | n.a. | D119G; Q121H; N129S; T131I; D133G; D141E; V169A (all **) | n.a. | D119G; Q121H; N129S; T131I; D133G; D141E; V169A (all **) |
|  | **Sites with dN/dS>1**  **(BEB analysis)** | n.a. | n.a. | n.a. | Q121H; N129S; D133G; D141E; V169A (not significant) | n.a. | Q121H; N129S; D133G; D141E; V169A (not significant) |
| **PROPIECE** | **Log likelihood** | -593.27 | -585.85 | -591.82 | -585.88 | -592.88 | -585.88 |
|  | **Parameters estimates** | ω=1.24385 | ω0=0.49, p0=0.76, ω1=3.33 p1=0.14, ω2=7.32 p2=0.1 | ω0= 0, p0=0.29, (ω1=1) p1=0.71 | ω0=0.45, p0=0.61, ω1=1 p1=0.21, ω2=5.96 p2=0.18 | p=0.012, q=0.005 | p0=0.81, p=5.23, p1=0.19, q= 3.99, ω=5.85 |
|  | **Sites with dN/dS>1**  **(NEB analysis)** | n.a. | N22I; W24R; L26Q; N30S; A31V; H33D*; P38YS*; D57E; T60IAS**; Q68E*; H76RD; L78S | n.a. | W24R; L26Q; N30S; H33D*; P38YS*; D57E; T60IAS**; Q68E*; H76RD; L78S | n.a. | N22I; W24R; L26Q; N30S; A31V; H33D*; P38YS*; D57E; T60IAS**; Q68E*; H76RD; L78S |
|  | **Sites with dN/dS>1**  **(BEB analysis)** | n.a. | n.a. | n.a. | W24R; L26Q; N30S; H33D; P38YS;T60IAS*; Q68E*; H76RD | n.a. | W24R; L26Q; N30S; H33D; P38YS;T60IAS*; Q68E*; H76RD; L78S |

**Table S7.** Statistical likelihood ratio tests comparing substitution models on BRICHOS and Propiece sequences. The deviances (LRT) calculated from paired CodeML models are compared with the critical values of chi-square asymptotic distribution with appropriate degrees of freedom.

| **BRICHOS** |  | **Models** | | |
| --- | --- | --- | --- | --- |
|  |  | **M0 versus M3** | **M1 versus M2** | **M7 versus M8** |
|  | deviance | 13.32 | 4.1 | 4.16 |
|  | df | 4 | 2 | 2 |
|  | p-value | **0.0357** | 0.1287 | 0.1249 |
| **PROPIECE** |  |  | **Models** |  |
|  |  | **M0 versus M3** | **M1 versus M2** | **M7 versus M8** |
|  | deviance | 14.84 | 11.88 | 14 |
|  | df | 4 | 2 | 2 |
|  | p-value | **0.0005** | **0.0026** | **0.0009** |

**References**

1 Tasiemski, A. *et al.* Characterization and function of the first antibiotic isolated from a vent organism: the extremophile metazoan *Alvinella pompejana*. *PLoS ONE* **9**, e95737, doi:10.1371/journal.pone.0095737 (2014).

2 Plouviez, S., Le Guen, D., Lecompte, O., Lallier, F. H. & Jollivet, D. Determining gene flow and the influence of selection across the equatorial barrier of the East Pacific Rise in the tube-dwelling polychaete Alvinella pompejana. *BMC Evol Biol* **10**, 220, doi:10.1186/1471-2148-10-220 (2010).

3 Smith, J. M. Analyzing the mosaic structure of genes. *J Mol Evol* **34**, 126-129 (1992).

4 Huson, D. H. & Bryant, D. Application of phylogenetic networks in evolutionary studies. *Mol Biol Evol* **23**, 254-267, doi:10.1093/molbev/msj030 (2006).

5 Nielsen, R. Molecular signatures of natural selection. *Annu Rev Genet* **39**, 197-218, doi:10.1146/annurev.genet.39.073003.112420 (2005).

6 Yang, Z. & Bielawski, J. P. Statistical methods for detecting molecular adaptation. *Trends Ecol Evol* **15**, 496-503 (2000).

7 Parmakelis, A. *et al.* Anopheles immune genes and amino acid sites evolving under the effect of positive selection. *PLoS One* **5**, e8885, doi:10.1371/journal.pone.0008885 (2010).

8 Hahn, M. W. Distinguishing Among Evolutionary Models for the Maintenance of Gene Duplicates. *Journal of Heredity* **100**, 605-617, doi:10.1093/jhered/esp047 (2009).
